# The incidence risk of breast and gynecological cancer by antidepressant use: A systematic review and dose–response meta-analysis of epidemiological studies involving 160,727 patients

**DOI:** 10.3389/fonc.2022.939636

**Published:** 2022-10-14

**Authors:** Yanjia Zhuang, Xiaogang Pang, Yuchen Qi, Tianshu Zhang, Guimao Cao, Heming Xue, Yifan Xu, Shuoxin Xie, Yifan Liu, Yinuo Wang, Yunxiao Li, Ying Xiong, Yuanyuan Li, Hui Shen

**Affiliations:** ^1^ Laboratory of Brain Science, Innovation Research Institute of Traditional Chinese Medicine, Shandong University of Traditional Chinese Medicine, Jinan, China; ^2^ Experimental Center, Shandong University of Traditional Chinese Medicine, Jinan, China; ^3^ School of health, Shandong University of Traditional Chinese Medicine, Jinan, China; ^4^ Department of Anesthesiology, Affiliated Hospital of Shandong University of Traditional Chinese Medicine, Jinan, China; ^5^ School of Medicine, Shandong University of Traditional Chinese Medicine, Jinan, China; ^6^ School of Acupuncture, Shandong University of Traditional Chinese Medicine, Jinan, China; ^7^ Innovation Research Institute of Traditional Chinese Medicine, Shandong University of Traditional Chinese Medicine, Jinan, China

**Keywords:** antidepressant, depression, breast cancer, gynecological cancer, incidence, meta-analysis, systematic review, dose–response analysis

## Abstract

**Background and objective:**

Antidepressants are widely prescribed to treat depression and anxiety disorders that may become chronic conditions among women. Epidemiological studies have yielded inconsistent results on the correlation between antidepressant use and the incidence risk of female breast and gynecological cancer, along with uncertain dose–response relationship. Therefore, we performed a systematic review and dose–response meta-analysis to investigate the association.

**Methods:**

Web of Science, Embase, PubMed, The Cochrane Library, and PsycINFO were systematically searched in January 2022, with no language limits. Random-effect models were used to calculate pooled effect sizes and 95% confidence intervals between studies. Linear and non-linear dose–response analyses were performed to evaluate the dose or duration of antidepressant use affecting the incidence risk of female breast and gynecological cancer. Further subgroup analyses were systematically performed by stratifying almost all study characteristics and important potential confounders, in order to further clarify and validate the important potential hypotheses regarding the biological mechanism underlying this association.

**Results:**

Based on a systematic literature search, 34 eligible studies (27 case–control studies and 7 cohort studies) involving 160,727 female breast and gynecological cancer patients found that antidepressant use did not increase the incidence risk of female breast and gynecological cancer (pooled OR: 1.01; 95% CI: 0.97, 1.04, *I*² = 71.5%, *p* < 0.001), and even decreased the incidence risk of ovarian cancer (pooled OR: 0.91; 95% CI: 0.83, 1, *I*² = 17.4%, *p* = 0.293). There were a non-linear dose–response relationship (*p* non-linearity < 0.05) between the duration of antidepressant use and incidence risk of female breast cancer, and an inverse linear dose–response relationship between antidepressant use and the incidence risk of gynecological cancer, specifically with an increase of cumulative defined daily dose or duration to a high level, like 25,550 doses (OR: 0.91, 95% CI: 0.85–0.98, *p* linearity < 0.05) or 4,380 days (OR: 0.82; 95% CI: 0.7, 0.96, *p* linearity < 0.05), compared to never antidepressant users.

**Conclusion:**

This systematic review and dose–response meta-analysis found that antidepressant use did not increase the incidence risk of female breast and gynecological cancer and even decreased the incidence risk of ovarian cancer, along with a non-linear or linear dose–response relationship.

**Systematic Review Registration:**

PROSPERO https://www.crd.york.ac.uk/PROSPERO/display_record.php?RecordID=313364, identifier CRD42022313364.

## Introduction

Female breast and gynecological cancers account for 18.7% of all reported new cancer cases in all cancer cases worldwide in 2020, representing 38.9% new incidence and approximately 27.9% mortality (1) in women. Current projections indicate that, by 2070, the worldwide number of new breast and cervix cases diagnosed reach 5.03 million annually (2).

Prevalence of antidepressant (AD) use has been rising for decades (3, 4), in parallel with increased diagnosis of mental disorders, expanding indications for use, and longer treatment duration (5–7). Furthermore, women use AD at a rate approximately twofold higher than men (6), as the incidence of depression is 70% higher among women (8). Approximately 23% of women have an episode of depression throughout life and high recurrences (9, 10).

Recently, preclinical *in vivo* studies from the US Food and Drug Administration (FDA) have found that 63.6% (7/11) of examined ADs were associated with carcinogenicity (11). Actually, since the early 1990s, several studies, in both tumor cell cultures and animal models, have raised a possible association between AD and cancer risk (12). Several potential hypotheses have been proposed regarding the biological mechanism underlying this association. First, the specific tricyclic ADs have been found to be genotoxic and carcinogenic by using somatic mutation and recombination test (SMART) in wing cells of *Drosophila melanogaster* because of the nitrogen atom at position 5 in the seven-membered ring of the tricyclic molecule (13). Second, fluoxetine, a selective serotonin reuptake inhibitor (SSRI), and amitriptyline, a tricyclic AD (TCA), have been shown to promote the growth of mammary tumors in animal models by binding to growth regulatory intracellular histamine receptors and perturbing cellular growth and differentiation (12, 14). Third, the more important finding is that the growth and normal function of these organs are controlled by gonadotropin and female sex hormones, and it has been postulated that these hormones have an important role in the development of cancer of these organs. Certain monoaminergic medications may play a role in breast and gynecological carcinogenesis by affecting the release of gonadotropins and prolactin (15–18), as the human cervix contains functional gonadotropin receptors as do other parts of female genital tract (19). Both estrogens and prolactin have been shown to increase the incidence of spontaneous and chemically induced mammary tumors in rodents (20, 21). Raised endogenous estrogen levels and exogenous estrogen use are associated with the risk of breast and endometrial cancer in humans (22, 23). In addition, higher plasma prolactin levels are associated with breast cancer risk in a prospective study (24). Furthermore, prolactin, as a tumor-promoter, has been shown to stimulate proliferative activity in the mammary gland, suppress apoptosis (normal process of cell self-destruction), and upregulate the BRCA1 (breast cancer 1) gene (25, 26). Furthermore, the results of subsequent epidemiologic studies suggest that AD prescriptions are associated with increased risk of hormone-related cancer, including breast, endometrial, ovarian cancer (relative risk [RR] :  1.8; 95% CI: 1.15–2.81) (27) and cervical cancer (odds ratio [OR]:  1.22, 95% CI :  1.03–1.43) (28). However, this relationship has not been validated subsequently by *in vivo* or *in vitro* studies (29, 30). Furthermore, the antiproliferative effects of AD use have been supported by subsequent studies (31–35). Moreover, the results of epidemiologic studies have been inconclusive and demonstrated high (27, 36–43), low (44–47), or no change in risk (28, 48–63). Thus, the association of breast and gynecological cancer risk with the use of AD is highly conflicted, leaving an open question that will impede clinical practice. Given the increasing and widespread usage of AD, even a small increase in cancer risk associated with their use could translate into a large number of cancer cases at the population level. Furthermore, ADs are commonly prescribed after the diagnosis of cancer, not only for the treatment of depression but also for pain management (64). On the other hand, if the inverse association between AD use and breast and gynecological cancer risk proves to be causal, this may have major implications for the indications and prescribing of ADs. Thus, the hypothesis that ADs could promote or inhibit breast and gynecological cancer growth has important implications in terms of the etiology and treatment of breast and gynecological cancer and certainly merits further investigation.

Although a few systemic reviews have been carried out to elaborate this association, they all have limitations in some aspects. Most of them have been published mostly before 2012 (65) and have no new evidence published recently (52, 66). Some of them have performed neither a formal meta-analysis (67–72) nor a non-linear or linear dose–response analysis (73), and some of them have either no subgroup analysis or only incomplete subgroup analysis (73). Some of them have only focused on single tumor sites, such as breast (73) or ovarian cancer, respectively (74). However, as the majority of gynecological cancer share morphological and molecular features and familial breast and ovarian cancers have common genetic predisposition (75), a systemic review is needed that puts all hormone-related cancers, such as endometrial (45, 49), corpus uteri, and cervical cancer (28), together as an integral part of hormone-related cancer in female patients.

Therefore, in this study, we performed a comprehensive systematic review and meta-analysis, and a comprehensive subgroup analysis stratified by almost all study characteristics and important potential confounders to investigate the association between AD use and the incidence risk of female breast and gynecological cancer. Moreover, we performed a dose–response meta-analysis to evaluate the dose or duration of AD use affecting the incidence risk of female breast and gynecological cancer, and further clarify and validate the several important potential hypotheses regarding the biological mechanism underlying this association.

## Methods

This meta-analysis was registered in the International Prospective Register of Systematic Reviews (PROSPERO; registration No.: CRD42022313364: https://www.crd.york.ac.uk/PROSPERO/display_record.php?RecordID=313364) and adhered to the Meta-analysis of Observational Studies in Epidemiology (MOOSE) checklist (**Supplementary Material Table 1**) and Preferred Reporting Items for Systematic Reviews and Meta-analyses (PRISMA) reporting guideline for all processes (76, 77).

### Search strategy

We searched the following electronic databases for studies published from their inception until January 2022: Web of Science, Embase, MEDLINE (PubMed), The Cochrane Library (CENTRAL), and PsycINFO. The search strategy was implemented using combined index terms (Medical Subject Headings, and Emtree) and free-text keywords, including (e.g., neoplasms or cancer) AND (e.g., antidepressive agents or anti-depress*) AND (e.g., morbidity or incidence or risk or occurrence) AND (e.g., case–control studies or cohort studies). A full description of the initial and supplementary search strategies is available in **Supplementary Material Table 2**. We combined search results using a bibliographic management tool (EndNote, version X9).

### Selection criteria

The included studies were limited to (1) observational studies that (2) explored the relationship between AD use and the incidence risk of female breast and gynecological cancer, and (3) provided maximum adjusted risk estimates [risk ratios (RRs), odds ratios (ORs), and hazard ratios (HRs) with 95% confidence intervals (CIs)] or data allowing the calculation of the risk estimates and 95% CIs, and (4) studies were conducted in humans. Exclusion criteria were as follows: studies assessing the relationship between AD use and recurrence and mortality of treated breast and gynecological cancer; studies lacking a control group; specified population (e.g., HIV-infected patient); non-peer-reviewed reports (e.g., oral presentations, posters, dissertations, and conference abstracts) and ongoing studies; and studies with insufficient information. Additionally, only the latest and/or complete one was used in the meta-analysis for duplicate articles.

### Data extraction

Two reviewers independently extracted and cross-checked the data from the included studies. The following details were presented in this review: first author’s name, year of publication, country, study design, participant number, age, type of ADs, exposure definition, exposure assessment, statistical indicators, outcome with the maximum covariate-adjusted ORs, RRs, HRs and 95% CIs, and adjusted/matched factors. All of the selected articles were examined by two researchers independently in terms of quality according to a Newcastle–Ottawa Scale (NOS; range 1–9 with 1–3 indicating low quality, 4–6 indicating moderate quality, and 7–9 indicating high quality) (78). Any conflicts were handled by consensus with a senior author.

### Statistical analysis

Given the lower than 10% incidence of cancer, we approximated RR and HR as OR when pooling the estimates across the studies (79). Summary ORs (along with their corresponding 95% CIs) were calculated by performing random-effect meta-analysis for the overall relationship between the AD use and incidence risk of female breast and gynecological cancer on comparisons of the ever AD use group versus the never AD use group, and forest plots were used to summarize the pooled estimates and effect sizes. The Cochrane *Q* test and *I*² statistic were employed to evaluate heterogeneity between studies, and *I*² > 50% was considered statistically significant. In order to examine its source, we performed subgroup analyses according to almost all relevant factors that may lead to significant heterogeneity.

Moreover, we used the G-L method (80) to carry out a dose–response meta-analysis using the levels of cumulative defined daily dose (81) (CDDD) or duration of AD use (days) and the adjusted natural log of the ORs with their SE. If the dose or duration was reported by range, we assigned the midpoint of the upper and lower boundaries in each category as the average duration or dose. If the highest category was open-ended, we considered the width of the category to be the same as that of the adjacent category. If the lowest category was open-ended, the lowest boundary was assumed to be zero. We included studies for this dose–response analysis only if they reported the distributions of cases and total persons or person-years, as well as the ORs and 95% CI with the variance estimates for at least three quantitative exposure categories. Step 1, we performed a non-linear dose–response meta-analysis by restricted cubic splines with three or four knots of the distribution, then based on the χ² and *p*-value calculated in step 1, we determined whether a linear (*p* > 0.05) or non-linear (*p* < 0.05) dose–response meta-analysis should be adopted.

The funnel plots and Egger’s test were used to detect potential publication bias (82). To assess the stability of the results, a leave-one-out sensitivity analysis was carried out. All statistical tests were two-sided using a significance level of *p* < 0.05. Analyses were performed with Stata 13.1 (StataCorp LP, College Station, TX, USA).

## Results

### Eligible studies and study characteristics

**Figure 1:**
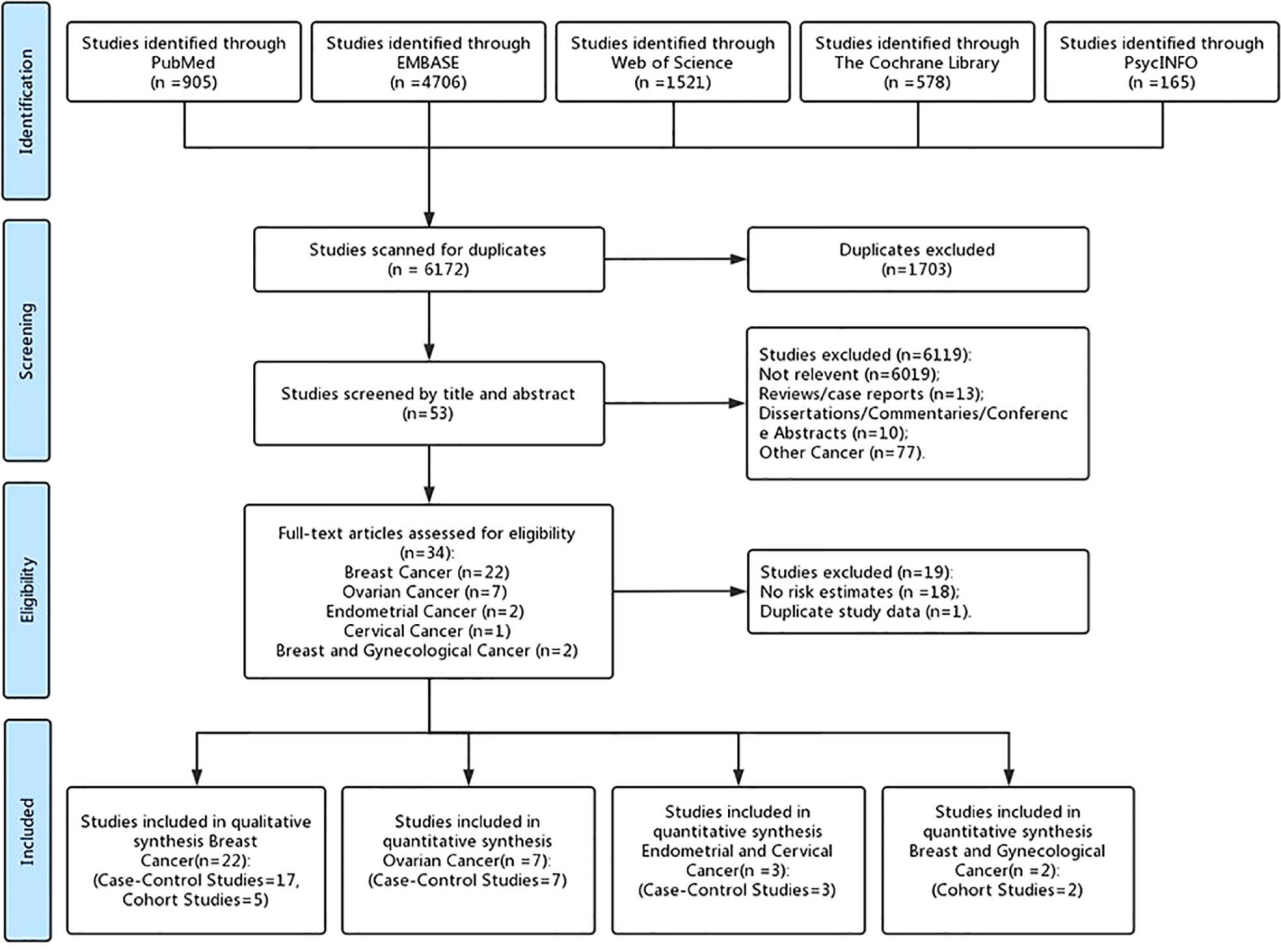
Flow diagram of included studies.

After identifying 7,875 references, 1,703 duplicate publications, 6,019 irrelevant studies, 13 reviews/case reports, 10 dissertations/commentaries/conference abstracts, and 77 other cancers were excluded after screening the titles and abstracts. The remaining 53 potentially studies were carefully read in full, and 18 of them were excluded for shorting the data of risk estimates and one was excluded for duplicate study data. Finally, 34 observational studies (27 case–control studies and 7 cohort studies) (27, 28, 36–57, 59–61, 63, 66, 83–87) were included in the present meta-analysis, including 22 female breast cancer studies, 7 ovarian cancer studies, 2 endometrial cancer studies, 1 cervical cancer study, and 2 female breast and gynecological cancer studies (**Figure 1**).

A total of 160,727 patients with female breast and gynecological cancer were involved, and the cases ranged in size from 20 to 45,147 participants. Overall, the studies were published between 1995 and 2021, involving participants from North America (*n* = 22), Europe (*n* = 8), and Asia (*n* = 4). Drug exposure was assessed and collected by questionnaires in 16 studies and databases in 18 studies, respectively. The quality of the included studies is assessed in **Supplementary Material Table 3**. The mean NOS score was 7 (median, 6.5; range, 5–8), indicating that the overall quality of articles was good. Additional characteristics of the included studies are shown in **Table 1**.

**Table 1:** Characteristics of observational studies of antidepressant drug use and breast and gynecological cancer risk.

First author, year, location	Cancer type	Study design	Study period	Participants (age)	Exposure definition	Exposure category (assessment)	Outcome indicators	Risk estimate	Adjusted/matched factors	NOS
Breast Cancer										
Reeves et al. (2018), USA	Breast	HP-CS	1992 -2013	2,667 invasive BC and 658 *in situ* BC in 66,692 NHS women and 1,347 invasive and 491 *in situ* BC in 89,820 NHSII women (age: >25)	Participants self-reported any current AD use	SSRI, other AD, SSRI, and other AD (questionnaires)	HR	No statistically significant associations were observed between SSRI use: HR = 0.92 (0.77–1.09) and invasive BC risk, ER+: HR = 0.89 (0.75–1.04), ER−: HR = 1.01 (0.73–1.39), postmenopausal HR = 0.93 (0.79–1.1).	Age, calendar year, BMI, count of clinical depression, age at menarche, current OC use (NHSII only), type of PMH use, age at menopause, age at first birth and parity, history of biopsy-confirmed benign breast disease, family history of BC, mammogram in prior 2 years, smoking status, physical activity, alcohol intake, AHEI score	7
Brown et al. (2016), USA	Breast	PP-CS	1993–1998	238 cases among 5,238 AD users/2,933 cases among 66,201 non-ADs users (age: 50–79)	Use of ADs for at least 2 weeks	TCAs, SSRIs, other ADs (questionnaire and Women’s Health Initiative Observational Study Database)	HR	AD use at baseline with risk of total BC: HR = 1.04 (0.92–1.20). Current AD use with increase of *in situ* BC: HR = 1.30 (0.99–1.75) after adjustment for depressive symptoms; this relationship was attenuated after adjustment for mammographic screening: HR = 1.08 (0.76–1.51)	Age, smoking status, alcohol use, parity, age at first birth, breastfeeding, oophorectomy, PMH use, age at menopause, race, physical activity, BMI, depression.	7
Chen et al. (2016), China	Breast	PR-CS	1999–2005	109 among 14 737 new AD female users/89 among 14,737 non-AD (age: ≥15)	At least 10 prescriptions and 1-year exposure to ADs	SSRIs, TCAs, other ADs (National Health and Research Institute Database in Taiwan)	OR/HR	AD prescription not associated with BC risk, 50+ AD prescriptions: OR = 0.97 (0.3-3.18)	Age, residence, insurance amount, and depressive disorder, AD prescriptions	8
Sun et al. (2015), Denmark	Breast	PR-CS	2003–2010	5,772 cases among 33,111 AD users/26,663 cases among 168,551 non-AD users (age: ≥18)	Current AD users refer to cancer patients who redeemed an AD prescription within 4 months before the cancer diagnosis. Former AD users refer to cancer patients who redeemed the last AD prescription more than 4 months before the cancer diagnosis.	SSRIs, TCAs, other ADs (Danish Cancer Registry Database, and Danish National Prescription Registry Database)	RR	Current AD users had a 32% higher 1-year mortality (MRR = 1.32, 95% CI: 1.29–1.35)	Sex, age, marital status, age at time of diagnosis, cancer stage, Charlson Comorbidity Index, marital status, education, calendar year	8
Haukka et al. (2010), Finland	Breast	PP-CS	1998–2005	1,925 BC cases, 218 ovarian cancer cases, and 302 Corpus uteri cancer cases among 418,588 AD users/1,780 BC cases, 255 ovarian cancer cases, and 370 Corpus uteri cancer cases among 418,588 non-AD users (age: 35–58)	Filled prescription at least once between 1998 and 2005; no AD use in previous 3 years	SSRIs, Other ADs (Finnish Cancer Registry and Social Insurance Institution Database)	RR	Highest cumulative exposure category of SSRI (over 4-year use) and BC: RR = 1.53 (1.14–2.03)	Age, sex, length of follow-up, cumulative use, primary site of cancer	6
Wang et al. (2001), USA	Breast	PR-CS	1989–1999	319 cases among 38,273 AD users/252 cases among 32,949 non-AD users (Age: ≥20)	Filled prescription for ADs from the index date to the end point (BC diagnosis, 1 July 1991, or death)	ADs, TCAs (Database: New Jersey Medical program, New Jersey Pharmaceutical Assistance to the Aged and Disabled, New Jersey Medicare, New Jersey Cancer Registry)	HR	ADs: HR = 1.04 (0.87–1.25); TCAs: HR = 1.09 (0.92–1.31)	Age, race, socioeconomic status, estrogen use, benign breast disease, obesity, Charlson Comorbidity Index, days in nursing home, alcohol abuse, and malignancies other than BC.	7
Busby et al. (2018), UK	Breast	HC-CS	1995–2010	45,147 cases/45,147 controls (average age: 62.8)	At least 1 prescription for the candidate medication, prescriptions in the year prior to the index date were excluded	Citalopram, Sertraline (Clinical Practice Research Datalink Database)	OR	Ever use citalopram: OR = 1.01 (0.96,1.07); ever use sertraline: OR = 1.01 (0.92,1.11)	Age, GP practice, year of diagnosis, comorbidities (AIDS, cerebrovascular disease, chronic pulmonary disease, congestive heart disease, dementia, diabetes, diabetes with complications, ductal carcinoma *in situ*, hemiplegia, mild liver disease, moderate liver disease, myocardial infarction, peptic ulcer disease, peripheral vascular disease, renal disease, and rheumatological disease), confounder medications (aspirin, digoxin, HRT, metformin, OC, and statin), deprivation, smoking status, alcohol consumption, and obesity	7
Boursi et al. (2015), UK	Breast	PC-CS	1995–2013	31,352 BC cases and 123,285 controls (average age: 62.5–62.8)	Any use of 1 of the 3 classes of ADs before index date, current users, with last prescription at least 6 months before index date, and past users with last prescription more than 6 months before index date	TCAs, SSRIs, SNRIs (Health Improvement Network Database)	OR	Current SSRI use with treatment initiation >1 year: OR = 1.12 (1.06–1.18) Initiation <1 year OR = 1.18 (1.08–1.29)	Obesity (BMI > 30), smoking, alcohol consumption, medical comorbidities including diabetes mellitus, HRT	7
Ashbury et al. (2012), Canada	Breast	PC-CS	2003–2007	2129 cases/21,297 controls (age: 28–79)	SSRI use within a 2-year period preceding the index date was excluded from the analysis.	SSRIs (Saskatchewan prescription Database)	OR	High or lower SSRI use not associated with increased risk of BC (OR = 1.01, CI = 0.88–1.17 and OR = 0.91, CI = 0.67– 1.25, respectively)	Age, marital status, income support status, residence status, OC use, HRT	6
Walker et al. (2011), UK	Breast	PC-CS	N/A	10,293 cases, 20,096 controls (mean age: 62.5)	Use of any TCA at least 1 year before the date of diagnosis of the index cancer	TCAs (GPRD)	OR	TCAs use with risk of BC: OR = 0.97 (0.91–1.04)	Age, smoking status, diagnosis of depression, alcohol use, and BMI.	6
Wernli et al. (2009), USA	Breast	PC-CS	2003–2006	2,908 cases/2,927 controls (age: 20–69)	Ever use of AD was defined as use for 3 months or more. Only exposures that occurred at least a year prior to the assigned reference date were included in analyses.	TCAs, SSRIs, SNRIs, NDRI, any AD (questionnaires)	OR	Ever use of ADs: OR = 0.89 (0.78–1.01); current use: OR = 0.92 (0.80–1.07; ever SSRI use: OR = 0.85 (0.72–1.00); ever TCA: OR = 0.90 (0.60–1.35); ever SNRI use: OR = 1.18 (0.72–1.92)	Age, year of interview, parity, age at first live birth, family history of BC, BMI, menopausal status, age at menopause, mammography, and type of HT.	6
Coogan et al. (2008), USA	Breast	HC-CS	1990–2006	820 invasive BC cases, and 2,852 controls (age: 25–79)	Regular use of SSRI: at least 4 days a week for at least 3 continuous months at least 1 year before admission	SSRIs (questionnaires)	OR	SSRI use among all invasive cancer: OR = 0.89 (0.62–1.29); among age ≥55: OR = 1.43 (0.76–2.69); among age ≥55:ER− (OR = 1.84, 95% CI: 0.66–5.16), PR− (OR = 1.85, 95% CI: 0.80–4.27), and ER−PR− (OR = 2.10, 95% CI: 0.73–6.02) tumors	Age, interview year, study center, race, OC use, HRT, years of education, religion, parity, age at first birth, menopausal status, family history of BC, age at menarche and alcohol consumption	6
Davis et al. (2007), USA	Breast	PC-CS	1992–1995, 2000–2001	549 cases/596 control (age: 20–74)	Regular use: at least 4 days/week for 6 months or longer, limited to the 10 years prior to diagnosis	ADs (questionnaires)	OR	Regular use: OR = 1.3 (0.8–1.9). 5–10 years of use: OR =1.4 (0.7–2.7). Within 2 years of diagnosis: OR = 1.3 (0.8-2.1). Postmenopausal: OR = 1.8 (1.0–3.0)	Age, parity, age at first pregnancy, family history of BC, early double oophorectomy, OC use, ever upper gastro-intestinal series, and ever smoker, mother/sister BC younger than age 45 and alcohol intake (if premenopausal); HRT (if postmenopausal)	6
Chien et al. (2006), USA	Breast	PC-CS	1997–1999	975 cases/1,007 controls (age: 65–79)	Ever use of an ADs in the category for at least 3 months	TCAs, SSRIs (questionnaires)	OR	Ever AD use: OR = 1.2 (0.9–1.6); TCA: OR = 1.2 (0.8–1.8); SSRI: OR = 1.2 (0.8–1.8); ever SSRI use and PR− BC: OR = 1.8 (1.1–3.6); ER+/PR− BC: OR = 2.0 (1.1–3.8)	Age at reference date and county of residence, race/ethnicity, income, marital status, education, time since last routine medical checkup, age at menarche, parity, age at first birth, type of menopause, age at menopause, duration of OC use, use of PMH, first degree family history of BC, cigarette smoking status, alcohol consumption, and BMI, history of various medical conditions such as depression, hypertension, hypercholesterolemia, arthritis, diabetes mellitus, and thyroid problems	7
Fulton-Kehoe et al. (2006), USA	Breast	PC-CS	1990–2001	2,904 cases/14,396 controls (age: 30–79)	Filled AD prescription at least twice in a 6-month interval at least 1 year before index date	TCAs, SSRIs, atypical ADs (Group Health Cooperative Database)	OR	Any AD: OR = 1.04 (0.94–1.16); TCA: OR = 1.06 (0.94–1.19); SSRI: OR = 0.98 (0.8–1.18); atypical: OR = 0.95 (0.78–1.16)	Age, length of enrollment, calendar year, family history of BC, parity/age at first birth, duration of HRT use, BMI, history of screening mammogram in 2 years prior to reference date.	7
Tamin et al. (2006), Canada	Breast	PC-CS	1981–2002	7,330 BC cases and 29,320 controls (age: 20.4–82.5)	Prescription database for the period between 1 January 1976 or the first coverage initiation date (whichever was later) and the index date. Exposure to TCAs in the year preceding the date of diagnosis was excluded from the analyses.	Genotoxic TCAs, non-genotoxic TCAs (Prescription Drug Plan Database)	RR	High doses of genotoxic TCAs 11–15 years before diagnosis: RR = 1.17 (95% CI: 0.79–1.74), high levels of non-genotoxic TCAs during the same period: RR = 0.95 (95% CI: 0.61–1.48).	Use of estrogen, OC, and NSAIDs, age	7
Coogan et al. (2005), USA	Breast	HC-CS	1988–2002	2,138 cases/2,858 controls (age: 24–73)	Regular use of SSRIs as use on at least 4 days per week for at least 3 continuous months. Any other use was considered sporadic, at least 1 year before admission	SSRIs (questionnaires)	OR	Regular use SSRI: OR = 1.1 (0.8–1.7); continued use: OR = 1.2 (0.8, 1.8); discontinued use: OR = 1.1 (0.5–2.6); 4 or more years: OR = 0.7 (0.5–1.5)	Age, study center and year of interview, alcohol consumption, religion, family history of BC, race, presence of benign breast disease, parity, menopausal status, age at menopause, age at first birth, BMI, and age at menarche.	5
Gonzalez- Perez et al. (2005), UK	Breast	PC-CS	1995–2001	3,708 cases/20,000 controls (age 30–79)	Drug exposure of study subjects at any time before the index date (including medication use recorded before the start date of the study)	SSRIs, TCAs, other ADs (GPRD)	OR	Current SSRI: OR = 0.98 (0.81–1.19); current TCA: OR = 0.86 (0.73–1.00); current other ADs: OR = 1.15 (0.82–1.61). 1 year SSRI: OR = 0.76 (0.53–1.09); 1 year TCA: OR = 0.87 (0.70–1.09)	Age, calendar year, depression, BMI, alcohol intake, HRT use, NSAID use, and prior benign breast disease.	6
Moorman et al. (2003), USA	Breast	PC-CS	1996–2000	938 invasive cases/771 controls; 507 carcinoma *in situ* cases/455 controls (age: 20–74)	Ever-users of antidepressants if they reported 3 or more months of use.	TCAs, SSRIs, atypical ADs, MAOI, lithium, or multiple types (questionnaires)	OR	Invasive BC: Any use of any ADs: OR = 1.0 (0.7–1.2); SSRI: OR = 1.0 (0.7–1.5); TCA: OR = 1.0 (0.7–1.5); multiple types: OR = 0.9 (0.6–1.3). Carcinoma *in situ* of the breast: Any use of any ADs: OR = 0.6 (0.4–0.8); SSRI: OR = 0.6 (0.4–0.9); TCA: OR = 0.4 (0.2–0.8); multiple types: OR = 0.9 (0.5–1.7)	Age, race, age at menarche, age at first full-term pregnancy, lactation history, menopausal status, family history of BC in a first-degree relative, OC use, HRT, educational level, BMI, waist-to-hip ratio, alcohol consumption, and smoking history.	6
Steingart et al. (2003), Canada	Breast	PC-CS	1996–1998	3,133 cases/3,062 controls (age: 25–74)	Taken daily for ≥2 months, started ≥12 months before diagnosis date for cases and referent date for controls	SSRIs, TCAs MAOIs, atypical ADs (questionnaires)	OR	Overall use of ADs: OR = 1.20 (0.96–1.51); SSRI: OR = 1.32 (0.97–1.80); TCA: OR = 1.10 (0.83–1.45); MAOI: OR = 0.80 (0.27–2.4); atypical: OR = 1.04 (0.5–2.16)	Age, height, BMI, age at menarche, parity, age at menopause, OC use, alcohol consumption, family history of BC, history of benign breast disease, clinical depression, anxiety.	7
Sharpe et al. (2002), Canada	Breast	PC-CS	1981–1995	5,882 cases/23, 517 controls (age: ≧̸35)	Filled prescription for TCA before the index date	The nongenotoxic TCAs, the genotoxic TCAs (Saskatchewan Prescription Drug Plan Database)	RR	Heavy exposure to any TCAs was associated with an elevated rate ratio for BC 11–15 years later (2.02, 95% confidence interval: 1.34–3.04)	Age and index date and adjusted for the effects of exposure during the other periods.	5
Cotterchio et al. (2000), Canada	Breast	PC-CS	1995–1996	701 cases/702 controls (age: 25–74)	Use of ADs for at least 2 weeks	TCAs, SSRIs, atypical medication use, MAOI (questionnaire)	OR	Any AD use: OR = 0.8 (0.5–1.4); TCA: use ≥25 months: OR = 2.1 (0.9–5.0); paroxetine: OR = 7.2 (0.9–58.3)	Age, age at menopause for SSRI, menopausal status, household income, history of clinical depression, BMI, family history of BC, and benign proliferative breast disease	5
Kelly et al. (1999), USA	Breast	HC-CS	1977–1996	5,814 cases/5,095 cancer controls and 5,814 non-cancer controls (age: 18–69)	Regular use: ≧4 days per week for ≧4 week excluding use begun <1 year before admission	SSRIs, TCAs, other ADs (questionnaire)	RR	TCA: cases vs. non-cancer controls: RR = 0.8 (0.6–1.0); SSRI: cases vs. non-cancer: RR = 1.5 (0.8–2.8)	Age, region, race, religion, year of interview, age at menarche, age at first birth, BMI, history of benign breast disease, menopausal status, history of BC in mother or sister, current alcohol consumption, and number of lifetime hospitalizations.	6
Ovarian Cancer										
Morch et al. (2017), Denmark	Ovarian	PC-CS	2000–2011	4,103 cases/58,706 controls (age: 30–84)	Two or more prescriptions on separate dates and non-use as fewer than two prescriptions. Use of antidepressants within 1 year prior to index date was disregarded	SSRIs, TCAs, others (National Prescription Registry Database)	OR	Use of any type of ADs: OR = 0.86 (0.79–0.95). SSRIs: OR = 0.85(0.74–0.96); TCA: OR = 0.99 (0.78–1.26); other ADs: OR = 1.05 (0.76-1.46)	Age, paracetamol, NA-NSAID, statins, aspirin, HT, HC, infertility, endometriosis, diabetes, COPD/asthma, hysterectomy, tubal ligation, parity, and education	7
Wu et al. (2015), China	Ovarian	PC-CS	1997–2011	957 cases/9,570 controls (age: 49.7 ± 15.1)	“Ever-used” indicated that a participant had received a prescription for any AD between 1 and 13 years before the index date. All drug exposure in the year immediately prior to the index date was excluded	ADs, SSRIs, TCAs, others (National Health Insurance Research Database)	OR	Ever use of AD: OR = 1 (0.84–1.18); SSRI only: OR = 1.3 (0.97–1.74); TCA: OR = 0.94 (0.78–1.14); other AD: OR = 0.84 (0.63–1.13)	Age, chronic renal failure, endometriosis, infertility, and mean number of hospitalizations per year	7
Moorman et al. (2005), USA	Ovarian	PC-CS	1999–2003	593 cases/628 controls (age: 20–74)	Asked if they had taken antidepressants for more than 6 months	Any use of AD, SSRI only, TCA only, other types of ADs, multiple types of AD. (questionnaire)	OR	Any use of AD: OR = 0.9 (0.7–1.2) > 10 years of use: OR = 0.7 (0.4–1.4); SSRI only: OR = 1.0 (0.7–1.5); >5 years: OR = 1.2 (0.5–2.2); TCA only: OR = 0.5 (0.2–1.1); Other AD: OR = 0.8 (0.4–1.5); Multiple types: OR = 0.9 (0.5–1.6).	Age, race, family history of ovarian cancer, number of full-term pregnancies, months of OC use, alcohol use, smoking, BMI, waist-to-hip ratio, and history of infertility, tubal ligation, or endometriosis had a minimal effect on the ORs.	6
Dublin et al. (2002), USA	Ovarian	PC-CS	1981–1997	314/790 (age: 35–79)	Ever use: ever filled two prescriptions for a drug in a particular class within a 6-month period. Continuous use: lasting at least 6 months and including at least two prescriptions, each prescription contained enough pills to last until the next prescription was filled, assuming daily use and 75% compliance. Filled prescription of AD in 1.5 years prior to the reference date	Serotonin alone or mixed with norepinephrine reuptake inhibitor, dopamine/norepinephrine reuptake inhibitors (Health maintenance organization Database)	OR	Overall use of AD: 2 prescriptions within 6 months: OR = 0.71 (0.47–1.10). Continuous use for over 6 months OR = 0.64 (0.36–1.10)	Age, length of Group Health Cooperative membership, and reference date	6
Coogan et al. (2000), USA	Ovarian	HC-CS	1976–1998	748 cases/1,496 cancer controls and 1,496 noncancer controls (age <80)	Regular use (at least 4 days/week for at least 1 month). Individuals whose use took place exclusively within the year before admission were kept in a separate category	SSRIs TCAs (questionnaire)	OR	The OR with noncancer controls for regular use of SSRIs was 3.0 (95% CI: 0.8 ± 10.5), but the multivariate OR was 0.7 (95% CI: 0.2 ± 2.2).	Age, study center, interview year, race, religion, smoking status, parity, age at menarche, age at menopause, OC use, BMI, and number of physician visits in the year prior to hospitalization.	5
Harlow et al. (1998), USA	Ovarian	PC-CS	1992–1997	563 cases/523 controls (median age: 49–50)	Continuous use of psychotropic drug for 6 months or longer, during at least 1 year prior to the index date	Serotonin alone or mixed with norepinephrine reuptake inhibitor, dopamine/norepinephrine reuptake inhibitors (questionnaire)	OR	Risk of epithelial ovarian cancer with serotonin alone or mixed with norepinephrine reuptake inhibitor: OR = 0.9 (0.5–1.8). Dopamine/norepinephrine reuptake inhibitors: OR = 2.4 (1.1–5.2)	Age, education, parity, OC use, cigarette smoking, center, marital status, and premenstrual symptomatology	6
Harlow et al. (1995), USA	Ovarian	PC-CS	1978–1987	450 cases/454 controls (age: 18–80)	Used any other medications on a regular basis for more than 1 month	AD (questionnaire)	OR	Prior use of antidepressants exceeding 1 month was associated with an increased risk of ovarian cancer: adjusted OR = 2.1 (0.9–4.8). The association was confined primarily to women whose first use occurred before age 50 years: adjusted OR = 3.5 (1.3–9.2)	Age, race, precinct of residence, parity, prior use of OC, religion, BMI, prior hysterectomy, and reported therapeutic abortion	6
Endometrial Cancer										
Sperling et al. (2021), Danish	Endometrial	PC-CS	2000–2016	8,164 cases/122,432 controls (age: 30–84)	Defined use as two or more filled prescriptions on separates dates. Non-use was defined as fewer than two filled prescriptions of any AD. Disregarded use within 1 year prior to the index date	SSRIs, TCAs, and other ADs (Danish nationwide health and demographic registries Database)	OR	SSRI use was associated with an OR of 0.88 (95% CI: 0.82–0.96) for endometrial cancer, whereas the association with TCA use was close to unity (OR 1.05, 95% CI: 0.90–1.22). Use of other antidepressants yielded an OR of 0.86 (95% CI: 0.71–1.03).	Age, obesity, diabetes, COPD, HRT, bisphosphonate, low-dose aspirin, parity and educational level.	8
Lin et al. (2016), China	Endometrial	PC-CS	1997–2008	8,392 cases/82,432 controls (age: >18)	AD prescription within 365 days before the index date.	TCAs, SSRIs, SNRIs (National Health Insurance Research Database)	OR	No association between endometrial cancer incidence and AD prescription, in either SSRIs (adjusted odds ratio [OR] = 0.98; 95% confidence interval [CI], 0.84–1.15) or SNRIs (adjusted OR = 1.14; 95% CI: 0.76–1.71) and higher cumulative doses of AD prescription	Income, urbanization, depressive disorder, anxiety disorder, type 2 diabetes mellitus, hypertension, hypercholesterolemia, obesity, estrogen, progesterone and estrogen in combination, aspirin, NSAID, statins use	7
Cervical Cancer										
Chan et al. (2015), China	Cervical	PC-CS	1997–2008	26,262 cases/129,490 controls (mean age: 55.5 ± 13.2)	Exposure was defined daily dose (DDD) defined by WHO, excluded antidepressants exposure in the year directly before the index date.	TCAs, MAOIs, SSRIs, SNRIs, SARI, NaSSa, and NDRI (National Health Insurance Research Database)	OR	An increased rate of cancer cases was associated with trazodone prescription, moderated by DDD, that is, for cumulative doses≧28 DDD, adjusted OR = 1.22, 95% CI : 1.03–1.43 and cumulative doses≧168 DDD, adjusted OR = 1.61, 95% CI : 1.14–2.28.	Age, income, urbanization, depressive disorders, type 2 DM, COPD, asthma, HIV infection, STD, Pap smear frequency, and aspirin use.	7
Breast/Ovarian/Endometrial Cancer										
Kato et al. (2000), USA	Breast/ovarian/endometrial	PP-CS	1985–1991	566 BC, 47 ovarian, and 67 endometrial cancers among 15,270 women	Use of ADs in 4 weeks preceding enrollment in the study	ADs (questionnaire)	RR	ADs and BC: RR = 1.75 (1.06–2.88); AD and breast/ovarian/endometrial cancers: RR = 1.80 (1.15–2.81); AD and premenopausal all hormone-related cancers: RR = 0.99 (0.47–2.09), postmenopausal: RR = 3.08 (1.76–5.38)	Age, Quetelet index, age at menarche, menopausal status, parity, and family history of BC.	7

HP-CS, hospital-based prospective cohort study; PP-CS, population-based prospective cohort study; PR-CS, population-based retrospective cohort study; HC-CS, hospital-based case–control study; PC-CS, population-based case–control study; HT/HRT, hormone therapy/hormone replacement therapy; N/A, not available; NA-NSAID, non-aspirin nonsteroidal anti-inflammatory drugs; OC/HC, oral contraceptive/hormonal contraceptives; NSAID, nonsteroidal anti-inflammatory drugs; BMI, body mass index; PMH, postmenopausal hormone; AHEI, Alternate healthy eating index; NOS, Newcastle–Ottawa scale; PR, progesterone receptor; ER, estrogen receptor; COPD, chronic obstructive pulmonary disease; AD, antidepressant; SSRIs, selective serotonin reuptake inhibitors; TCAs, tricyclic antidepressants; MAOIs, monoamine oxidase inhibitors; SNRIs, serotonin and norepinephrine reuptake inhibitors; NaSSas, noradrenergic and specific serotonergic antidepressants; SARIs, serotonin antagonist and reuptake inhibitors; NDRIs, norepinephrine and dopamine reuptake inhibitors; BC, breast cancer; GPRD, general practice research database; HR, hazard ratio; OR, odds ratio; RR, relative risk.

### Meta-analysis of ever vs. never antidepressant medication use and incidence risk of female breast and gynecological cancer

**Figure 2:**
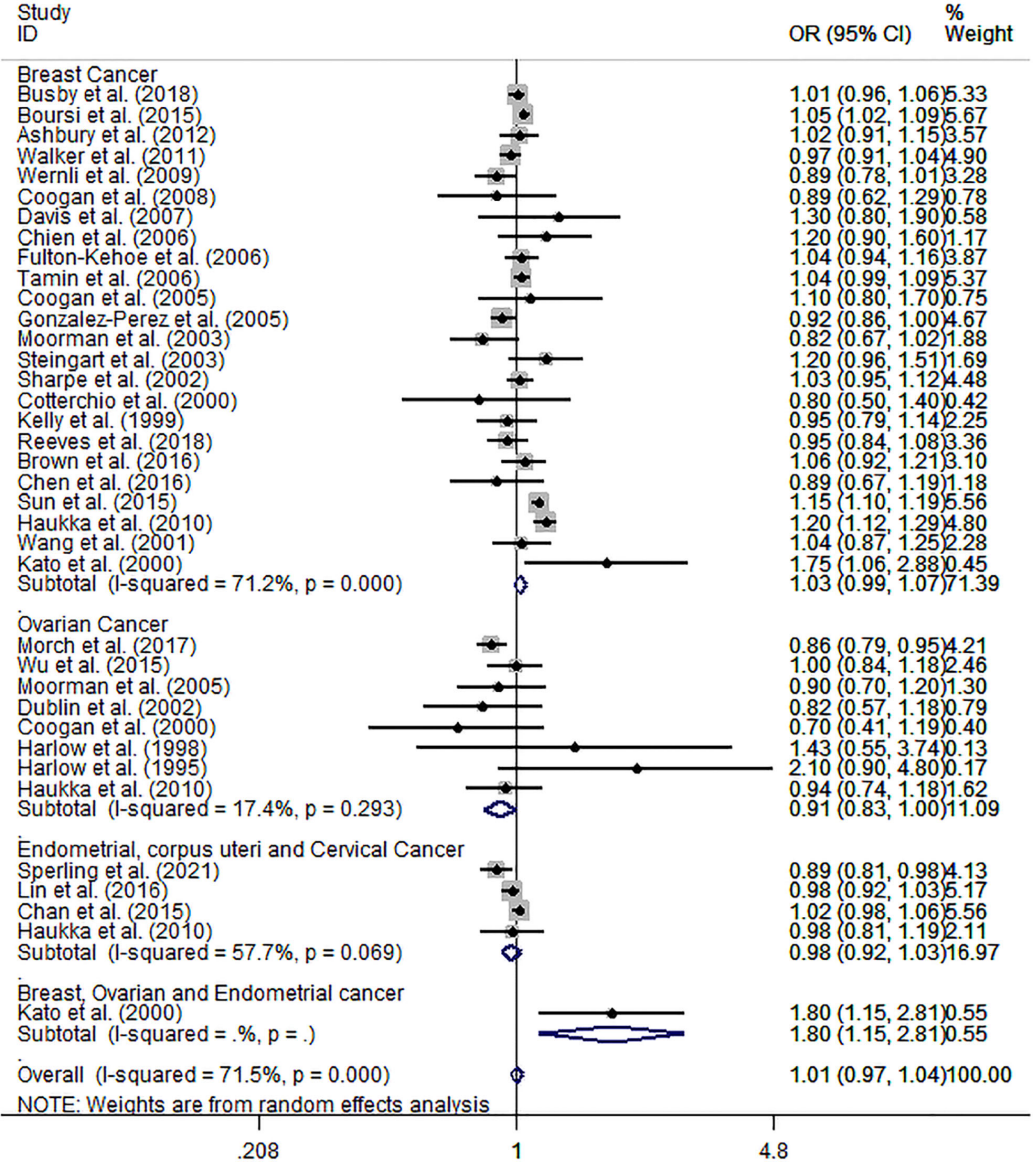
Forest plot of ever vs. never antidepressant use and incidence risk of breast and gynecological cancer.

The meta-analysis results of 34 epidemiological studies showed that ever AD use was unrelated to overall incidence risk of breast and gynecological cancer (pooled OR: 1.01; 95% CI: 0.97, 1.04), with a high heterogeneity (*I*² = 71.5%, *p* < 0.001). Furthermore, subgroup analyses for specific cancer sites showed a slightly decreased incidence risk of ovarian cancer (pooled OR: 0.91; 95% CI: 0.83, 1, *I*² = 17.4%, *p* = 0.293); no significant relation with endometrial, corpus uteri, and cervical cancers (pooled OR: 0.98; 95% CI: 0.92, 1.03, *I*² = 57.7%, *p* = 0.069); and a weakly increased incidence risk of female breast cancer (pooled OR: 1.03; 95% CI: 0.99, 1.07), with a high heterogeneity (*I*² = 71.2%, *p* < 0.001), compared to never AD users (**Figure 2**).

In the light of the remarkable between-study heterogeneity, we investigated its potential sources by subgroup analyses of the study characteristics and confounders. The detailed results showed in **Table 2**. The results showed that the incidence risk of female breast and gynecological cancer was not statistically different across great majority strata, but the incidence risk appeared to be stronger in exposures that occurred less than 1 year prior to the index date (pooled OR: 1.25; 95% CI: 0.56, 2.8) than that in exposures that occurred at least 1 year prior to the index date (pooled OR: 0.89; 95% CI: 0.81, 0.97), and stronger in premenopausal female patients (pooled OR: 1.62; 95% CI: 0.42, 6.21) than that in postmenopausal female patients (pooled OR: 0.93; 95% CI: 0.79, 1.09) for ovarian cancer. It also appeared to be stronger in estrogen receptor (ER)+ progesterone receptor (PR)− (pooled OR: 1.47; 95% CI: 0.94, 2.3), PR− (pooled OR: 1.3; 95% CI: 0.75, 2.26), ER−PR− (pooled OR: 1.25; 95% CI: 0.77, 2.02), and ER− (pooled OR: 1.15; 95% CI: 0.94, 1.42) than that in ER+ (pooled OR: 1; 95% CI: 0.93, 1.07), PR+ (pooled OR: 1.01; 95% CI: 0.78, 1.31), and ER+PR+ (pooled OR: 1.02; 95% CI: 0.79, 1.32) for breast cancer.

**Table 2:** Subgroup analyses of the study characteristics and confounders.

Breast Cancer						Ovarian Cancer					Endometrial, Corpus Uteri, and Cervical Cancer					Summary				
Subgroup	No. of studies	OR 95% CI	*I* ^2^ (%)^a^	Ph^b^	Ph^c^	No. of studies	OR 95% CI	*I* ^2^ (%)^a^	Ph^b^	Ph^c^	No. of studies	OR 95% CI	*I* ^2^ (%)^a^	Ph^b^	Ph^c^	No. of studies	OR 95% CI	*I* ^2^ (%)^a^	Ph^b^	Ph^c^
Type of studies					0.048					0.81					0.918					0.043
Case–control	17	1 (0.97,1.04)	43.2	0.03		7	0.91 (0.81,1.02)	27.7	0.217		3	0.97 (0.96,1.02)	71.7	0.029		27	0.99 (0.96,1.02)	53.1	0.001	
Cohort	7	1.09 (1.01,1.18)	66.7	0.006		1	0.94 (0.74,1.19)	N/A	N/A		1	0.98 (0.81,1.19)	N/A	N/A		7	1.08 (1,1.17)	66.4	0.002	
Type of control subjects					0.219					0.323										0.746
Population based	19	1.04 (0.99,1.09)	75.2	<0.001		7	0.92 (0.83,1.01)	21.3	0.269		4	0.98 (0.92,1.03)	57.7	0.069		28	1.01 (0.97,1.05)	75.1	<0.001	
Hospital based	5	1 (0.96,1.04)	0	0.786		1	0.7 (0.41,1.19)	N/A	N/A		0	N/A	N/A	N/A		6	1 (0.95,1.04)	0	0.636	
Geographic location					0.48					0.358					0.031					0.888
Europe	6	1.05 (0.98,1.12)	90.5	<0.001		2	0.87 (0.8,0.95)	0	0.487		2	0.91 (0.83,0.99)	0	0.379		8	1 (0.94,1.07)	89.3	<0.001	
Asia	1	0.89 (0.67,1.19)	N/A	N/A		1	1 (0.84,1.19)	N/A	N/A		2	1.01 (0.97,1.04)	23.1	0.254		4	1.01 (0.97,1.04)	0	0.572	
North America	17	1.02 (0.97,1.06)	26.6	0.149		5	0.93 (0.71,1.21)	32.6	0.204		N/A	N/A	N/A	N/A		22	1.02 (0.97,1.07)	38	0.034	
Exposure assessment					0.449					0.499					N/A					0.715
Database	12	1.04 (0.99,1.09)	80.2	<0.001		4	0.89 (0.83,0.96)	0	0.433		4	0.98 (0.92,1.03)	57.7	0.069		18	1.01 (0.97,1.05)	0.794	<0.001	
Questionnaire	12	1 (0.92,1.1)	43.6	0.053		4	1.02 (0.69,1.5)	46	0.135		N/A	N/A	N/A	N/A		16	1.03 (0.93,1.13)	50.3	0.009	
Only exposures that occurred at least a year prior to the index date					0.868					0.411					0.782					0.517
Yes	10	1.01 (0.98,1.04)	0.113	0.339		5	0.89 (0.81,0.97)	5.5	0.375		2	0.96 (0.84,1.1)	85.1	0.009		17	0.98 (0.95,1.02)	48.5	0.013	
No	7	1.02 (0.91,1.14)	79	<0.001		2	1.25 (0.56,2.8)	72	0.059		1	0.98 (0.93,1.04)	N/A	N/A		10	1.01 (0.93,1.1)	74.4	<0.001	
Number of cases					1					0.925					0.921					0.855
<800	7	1.03 (0.92,1.14)	33.1	0.175		6	0.92 (0.77,1.1)	16.7	0.306		1	0.98 (0.81,1.19)	N/A	N/A		13	1.02 (0.92,1.12)	39.3	0.059	
≥800	17	1.03 (0.98,1.07)	77.2	<0.001		2	0.91 (0.79,1.05)	57.2	0.126		3	0.97 (0.91,1.04)	71.1	0.029		22	1.01 (0.97,1.05)	79.5	<0.001	
Study quality scores					0.253					0.925					0.921					0.337
NOS > 6	13	1.05 (1,1.09)	68.4	<0.001		2	0.91 (0.79,1.05)	57.2	0.126		3	0.97 (0.91,1.04)	71.7	0.029		18	1.02 (0.98,1.06)	76.9	<0.001	
NOS ≤ 6	11	0.99 (0.90,1.08)	74.6	<0.001		6	0.92 (0.77,1.1)	16.7	0.306		1	0.98 (0.81,1.19)	N/A	N/A		16	0.98 (0.91,1.05)	64	<0.001	
Types of antidepressant drugs					0.197					0.57					0.02					0.733
ADs	6	1.12 (1.04,1.21)	32.2	0.194		3	1.02 (0.75,1.38)	51.7	0.126		1	0.81 (0.73,0.89)	N/A	N/A		10	1.09 (0.96,1.24)	82.7	<0.001	
TCAs	14	1.01 (0.97,1.04)	25.1	0.184		4	0.92 (0.79,1.07)	6.1	0.362		3	0.98 (0.91,1.06)	46	0.157		21	1 (0.97,1.03)	23.9	0.184	
SSRIs	17	1.02 (0.96,1.08)	60.4	0.001		5	0.97 (0.8,1.16)	44.3	0.127		4	0.96 (0.89,1.04)	56.4	0.076		24	0.99 (0.95,1.04)	65	<0.001	
MAOIs	1	0.8 (0.27,2.39)	N/A	N/A		N/A	N/A	N/A	N/A		2	0.99 (0.93,1.05)	0	0.631		3	0.99 (0.93,1.04)	0	0.829	
SNRIs	2	1.16 (0.91,1.47)	0	0.928		1	1.14 (0.61,2.13)	N/A	N/A		2	1.09 (0.92,1.29)	0	0.917		5	1.11 (0.97,1.27)	0	0.996	
SARIs	2	0.92 (0.77,1.11)	0	0.415		N/A	N/A	N/A	N/A		2	1.06 (0.73,1.55)	86.4	0.007		4	0.99 (0.78,1.24)	76.8	0.005	
NaSSas	N/A	N/A	N/A	N/A		1	1.25 (0.89,1.76)	N/A	N/A		2	0.82 (0.37,1.83)	70.9	0.064		3	1.01 (0.65,1.56)	57.5	0.095	
NDRIs	2	0.98 (0.69,1.39)	0	0.508		N/A	N/A	N/A	N/A		2	0.83 (0.29,2.35)	41.8	0.19		4	0.97 (0.71,1.33)	0	0.536	
Genotoxic or nongenotoxic TCA					1					0.31					N/A					1
Genotoxic TCA	8	1.04 (0.98,1.09)	0	0.81		1	1.38 (0.78,2.45)	N/A	N/A		N/A	N/A	N/A	N/A		9	1.04 (0.99,1.1)	0	0.792	
Nongenotoxic TCA	8	1.04 (0.97,1.12)	40.7	0.107		1	0.99 (0.74,1.32)	N/A	N/A		N/A	N/A	N/A	N/A		9	1.04 (0.97,1.11)	33	0.154	
The degree of serotonin reuptake inhibition of SSRIs					0.413					N/A					N/A					0.413
High inhibitors	8	1.04 (0.96,1.14)	0	0.616		N/A	N/A	N/A	N/A		N/A	N/A	N/A	N/A		8	1.04 (0.96,1.14)	0	0.616	
Lower inhibitors	1	0.91 (0.67,1.24)	N/A	N/A		N/A	N/A	N/A	N/A		N/A	N/A	N/A	N/A		1	0.91 (0.67,1.24)	N/A	N/A	
Different drug mechanism					N/A					0.234					N/A					0.234
Serotonin alone or mixed with norepinephrine reuptake inhibitor	N/A	N/A	N/A	N/A		3	0.97 (0.83,1.13)	0	0.779		N/A	N/A	N/A	N/A		3	0.97 (0.83,1.13)	0	0.779	
Dopamine/norepinephrine reuptake inhibitors	N/A	N/A	N/A	N/A		3	1.38 (0.79,2.42)	46.9	0.152		N/A	N/A	N/A	N/A		3	1.38 (0.79,2.42)	46.9	0.152	
Menopausal status (50/55 years of age)					0.054					0.423					0.761					0.885
Premenopausal or <age 50/55	4	0.84 (0.69,1.01)	33.5	0.211		2	1.62 (0.42,6.21)	85.5	0.009		1	0.89 (0.69,1.15)	N/A	N/A		8	1.02 (0.79,1.33)	77.3	0	
Postmenopausal or ≥age 50/55	6	1.03 (0.95,1.12)	22.6	0.264		2	0.93 (0.79,1.09)	0	0.483		1	0.93 (0.82,1.05)	N/A	N/A		10	1 (0.94,1.06)	74	0.374	
Types of breast cancer					0.73					N/A					N/A					0.73
Invasive breast cancer	3	0.97 (0.89,1.07)	0	0.861		N/A	N/A	N/A	N/A		N/A	N/A	N/A	N/A		3	0.97 (0.89,1.07)	0	0.861	
* In situ* breast cancer	3	1.08 (0.59,1.97)	87	<0.001		N/A	N/A	N/A	N/A		N/A	N/A	N/A	N/A		3	1.08 (0.59,1.97)	87	<0.001	
Hormone receptor status for breast cancer					0.477					N/A					N/A					0.128
ER+	5	1 (0.93,1.07)	0	0.456		N/A	N/A	N/A	N/A		N/A	N/A	N/A	N/A		5	1 (0.93,1.07)	0	0.456	
ER-	5	1.15 (0.94,1.42)	42.9	0.136		N/A	N/A	N/A	N/A		N/A	N/A	N/A	N/A		5	1.15 (0.94,1.42)	42.9	0.136	
PR+	2	1.01 (0.78,1.31)	0	0.36		N/A	N/A	N/A	N/A		N/A	N/A	N/A	N/A		2	1.01 (0.78,1.31)	0	0.36	
PR-	2	1.3 (0.75,2.26)	66.4	0.084		N/A	N/A	N/A	N/A		N/A	N/A	N/A	N/A		2	1.3 (0.75,2.26)	66.4	0.084	
ER+PR+	2	1.02 (0.79,1.32)	0	0.407		N/A	N/A	N/A	N/A		N/A	N/A	N/A	N/A		2	1.02 (0.79,1.32)	0	0.407	
ER+PR-	2	1.47 (0.94,2.3)	6.9	0.3		N/A	N/A	N/A	N/A		N/A	N/A	N/A	N/A		2	1.47 (0.94,2.3)	6.9	0.3	
ER-PR-	2	1.25 (0.77,2.02)	27.7	0.24		N/A	N/A	N/A	N/A		N/A	N/A	N/A	N/A		2	1.25 (0.77,2.02)	27.7	0.24	
With depressive symptoms					0.486					N/A					N/A					0.486
Yes	1	0.94 (0.63,1.4)	N/A	N/A		N/A	N/A	N/A	N/A		N/A	N/A	N/A	N/A		1	0.94 (0.63,1.4)	N/A	N/A	
No	1	1.1 (0.91,1.33)	N/A	N/A		N/A	N/A	N/A	N/A		N/A	N/A	N/A	N/A		1	1.1 (0.91,1.33)	N/A	N/A	
Adjustment for potential confounders																				
Age					<0.001					N/A					N/A					<0.001
Yes	23	1.02 (0.98,1.06)	59.7	<0.001		8	0.91 (0.83,1)	17.4	0.293		4	0.98 (0.92,1.03)	57.7	0.069		33	1 (0.97,1.08)	62	<0.001	
No	1	1.15 (1.1,1.19)	N/A	N/A		N/A	N/A	N/A	N/A		N/A	N/A	N/A	N/A		1	1.15 (1.1,1.19)	N/A	N/A	
Family history of breast/ovarian/endometrial/cervical cancer					0.419					0.883					N/A					0.842
Yes	12	1 (0.92,1.09)	43.4	0.054		1	0.9 (0.69,1.18)	N/A	N/A		N/A	N/A	N/A	N/A		13	1.02 (0.93,1.11)	51.5	0.013	
No	12	1.04 (1,1.09)	80.1	<0.001		7	0.92 (0.82,1.03)	29.2	0.206		4	0.98 (0.92,1.03)	57.7	0.069		21	1.01 (0.97,1.05)	77.4	<0.001	
HRT/PMH/OC/HC use					0.309					0.645					0.104					0.059
Yes	15	1.01 (0.97,1.05)	46	0.026		5	0.91 (0.75,1.09)	33.9	0.195		2	0.94 (0.86,1.03)	65.6	0.088		22	0.98 (0.95,1.02)	59.9	<0.001	
No	9	1.06 (0.98,1.16)	77.9	<0.001		3	0.96 (0.84,1.09)	0	0.615		2	1.02 (0.98,1.06)	0	0.69		12	1.05 (0.98,1.11)	75	<0.001	
Smoking status					0.431					0.739					N/A					0.77
Yes	8	1.01 (0.97,1.06)	47.2	0.066		3	0.88 (0.7,1.11)	0	0.423		N/A	N/A	N/A	N/A		11	1 (0.96,1.05)	39.6	0.085	
No	16	1.04 (0.98,1.1)	74.8	<0.001		5	0.92 (0.82,1.05)	40.6	0.151		4	0.98 (0.92,1.03)	57.7	0.069		23	1.01 (0.96,1.06)	77.1	<0.001	
Alcohol intake					0.14					0.883					N/A					0.772
Yes	14	1 (0.96,1.04)	45	0.035		1	0.9 (0.69,1.18)	N/A	N/A		N/A	N/A	N/A	N/A		15	1 (0.96,1.04)	42.6	0.041	
No	10	1.06 (0.99,1.13)	76.3	<0.001		7	0.92 (0.82,1.03)	29.2	0.206		4	0.98 (0.92,1.03)	57.7	0.069		19	1.01 (0.96,1.07)	78.7	<0.001	
Menopausal status/age at menarche					0.334					0.323					N/A					1
Yes	11	0.99 (0.91,1.09)	44	0.057		1	0.7 (0.41,1.19)	N/A	N/A		N/A	N/A	N/A	N/A		12	1.01 (0.92,1.12)	54.7	0.009	
No	13	1.04 (1,1.09)	78.7	<0.001		7	0.92 (0.83,1.01)	21.3	0.267		4	0.98 (0.92,1.03)	57.7	0.069		22	1.01 (0.97,1.05)	76.4	<0.001	
Parity/pregnancy/breastfeeding					0.516					0.645					0.015					0.48
Yes	12	1.01 (0.94,1.09)	43.9	0.051		5	0.91 (0.75,1.09)	33.9	0.195		1	0.89 (0.81,0.98)	N/A	N/A		18	0.99 (0.92,1.07)	58	0.001	
No	12	1.04 (0.99,1.09)	80.5	<0.001		3	0.96 (0.84,1.09)	0	0.615		3	1.01 (0.97,1.04)	0	0.503		16	1.02 (0.99,1.06)	74.7	<0.001	
Previous radiation for breast cancer					0.119					N/A					N/A					0.119
Yes	3	0.96 (0.88,1.06)	42.8	0.174		N/A	N/A	N/A	N/A		N/A	N/A	N/A	N/A		3	0.96 (0.88,1.06)	42.8	0.174	
No	21	1.04 (1,1.08)	71.8	<0.001		N/A	N/A	N/A	N/A		N/A	N/A	N/A	N/A		21	1.04 (1,1.08)	71.8	<0.001	
BMI/obesity					0.036					0.682					0.104					0.274
Yes	16	1 (0.96,1.04)	52.3	0.008		3	0.98 (0.62,1.54)	58.1	0.092		2	0.94 (0.86,1.03)	65.6	0.088		21	0.99 (0.95,1.03)	58.6	<0.001	
No	8	1.08 (1.02,1.15)	70.7	0.001		5	0.89 (0.83,0.96)	0	0.453		2	1.02 (0.98,1.06)	0	0.69		13	1.03 (0.97,1.09)	77	<0.001	
Depression					0.071					N/A					0.031					0.514
Yes	8	0.98 (0.92,1.04)	29.8	0.19		N/A	N/A	N/A	N/A		2	1.01 (0.97,1.04)	23.1	0.254		10	0.99 (0.95,1.03)	32.6	0.147	
No	17	1.05 (1,1.09)	72	<0.001		8	0.91 (0.83,1)	17.4	0.293		2	0.91 (0.83,0.99)	0	0.379		24	1.01 (0.97,1.06)	74.2	<0.001	
Infertility					N/A					0.865					N/A					0.613
Yes	N/A	N/A	N/A	N/A		4	0.94 (0.8,1.09)	52.7	0.096		N/A	N/A	N/A	N/A		4	0.94 (0.8,1.09)	52.7	0.096	
No	N/A	N/A	N/A	N/A		3	0.92 (0.76,1.12)	0	0.541		4	0.98 (0.92,1.03)	57.7	0.069		6	0.98 (0.93,1.02)	32.8	0.178	
Hysterectomy/oophorectomy					N/A					0.54					0.015					0.002
Yes	N/A	N/A	N/A	N/A		5	0.90 (0.77,1.06)	27.4	0.239		1	0.89 (0.81,0.98)	N/A	N/A		6	0.88 (0.82,0.95)	10.7	0.347	
No	N/A	N/A	N/A	N/A		3	0.96 (0.84,1.09)	0	0.449		3	1.01 (0.97,1.04)	0	0.503		6	1 (0.97,1.03)	0	0.63	
Other drugs use (aspirin/paracetamol/NSAID/statins)					0.21					0.202					0.921					0.065
Yes	3	0.99 (0.94,1.06)	72.4	0.027		1	0.86 (0.78,0.94)	N/A	N/A		3	0.97 (0.91, 1.04)	71.7	0.029		7	0.97 (0.93,1.01)	75.4	<0.001	
No	21	1.04 (0.99,1.09)	68.4	<0.001		7	0.95 (0.84,1.07)	8.4	0.364		1	0.98 (0.81,1.19)	N/A	N/A		27	1.03 (0.98,1.08)	63.6	<0.001	
Medical comorbidities (diabetes/COPD/chronic renal failure)					0.532					0.925					0.921					0.357
Yes	4	1.04 (1.01,1.07)	0	0.457		2	0.91 (0.79,1.05)	57.2	0.126		3	0.97 (0.91,1.04)	71.7	0.029		9	0.99 (0.95,1.03)	69.9	0.001	
No	20	1.02 (0.97,1.08)	75.2	<0.001		6	0.92 (0.77,1.1)	16.7	0.306		1	0.98 (0.81,1.19)	N/A	N/A		25	1.02 (0.97,1.07)	70.6	<0.001	

HT/HRT, hormone therapy/hormone replacement therapy; N/A, not available; NA-NSAID, non-aspirin nonsteroidal anti-inflammatory drugs; OC/HC, oral contraceptive/hormonal contraceptives; NSAID, nonsteroidal anti-inflammatory drugs; BMI, body mass index; PMH, postmenopausal hormone; NOS, Newcastle–Ottawa scale; PR, progesterone receptor; ER, estrogen receptor; COPD, chronic obstructive pulmonary disease; ADs, antidepressants; SSRIs, selective serotonin reuptake inhibitors; TCAs, tricyclic antidepressants; MAOIs, monoamine oxidase inhibitors; SNRIs, serotonin and norepinephrine reuptake inhibitors; NaSSas, noradrenergic and specific serotonergic antidepressants; SARIs, serotonin antagonist and reuptake inhibitors; NDRIs, norepinephrine and dopamine reuptake inhibitors; BC, breast cancer; OR, odds ratio; CI, confidence interval.

a I^2^ statistic was used to quantify the magnitude of between-study heterogeneity, and assigned values of 50% or less, 51–75%, and 76% or more for low, moderate, and high heterogeneity, respectively.

b p value for heterogeneity within each subgroup. A two-tailed p < 0.05 was considered statistically significant.

c p value for heterogeneity between subgroups with meta-regression analysis. A two-tailed p < 0.05 was considered statistically significant.

### Dose–response meta-analysis between the CDDD or duration of antidepressant use and incidence risk of female breast and gynecological cancer

**Figure 3:**
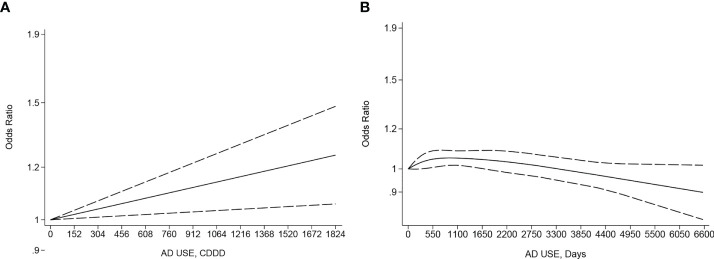
**(A)** Dose–response for cumulative defined daily dose (CDDD) of antidepressant (AD) use and incidence risk of breast cancer. **(B)** Dose–response for duration of antidepressant use and incidence risk of breast cancer. The black solid line and the black long dashed line represent the estimated odds ratios (ORs) with corresponding 95% confidence intervals (CIs) for the non-linearity or the linearity.

There was a significant linear dose–response association between the CDDD of AD use and incidence risk of female breast cancer in two eligible studies (66, 86) involving 48,852 cases and 2,141,294 participants (*p* linearity < 0.05, **Figure 3A**). The OR kept a slightly increasing trend without breaking 1.2 until the CDDD increased to 1,520. By comparison, a non-linear dose–response meta-analysis with 13 eligible studies (37, 38, 42, 47, 48, 55–57, 60, 61, 66, 83, 84) involving 109,215 cases and 398,024 controls (p non-linearity < 0.05, **Figure 3B**) showed a very slight increase in incidence risk of female breast cancer along with the increase of duration of AD use until 1,460 days (OR: 1.05; 95% CI: 1.01, 1.09), then followed a subsequent statistically significant inverse trend, specifically when an increase of duration to 6,570 days (OR: 0.9, 95% CI:0.79–1.02), compared to never AD users.

**Figure 4:**
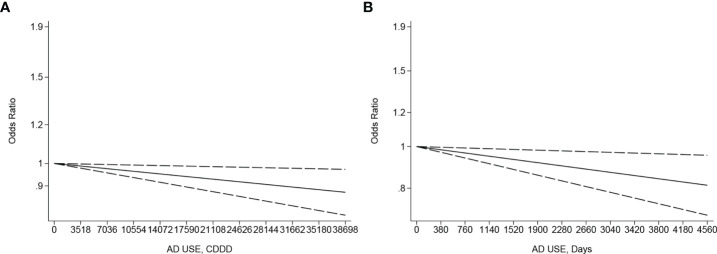
**(A)** Dose–response for CDDD of antidepressant use and incidence risk of ovarian, endometrial, and cervical cancer. **(B)** Dose–response for duration of antidepressant use and incidence risk of ovarian, endometrial, and cervical cancer. The black solid line and the black long dashed line represent the estimated odds ratios (ORs) with corresponding 95% confidence intervals (CIs) for the non-linearity or the linearity.

A negative linearity association existed between the CDDD or duration of AD use and the incidence risk of ovarian, endometrial, and cervical cancer, specifically with an increase of CDDD or duration to a high level, like 25,550 CDDD (OR: 0.91, 95% CI: 0.85–0.98, *p* linearity < 0.05, **Figure 4A**) or 4,380 days (OR: 0.82; 95% CI: 0.7, 0.96, *p* linearity < 0.05, **Figure 4B**) from four eligible studies (28, 45, 49, 86) involving 38,843 cases and 5,709,516 controls and eight eligible studies (36, 44–46, 49, 50, 59, 87) with 23,201 cases and 289,483 participants, respectively. Another negative linearity association also existed between the CDDD and the incidence risk of endometrial, corpus uteri, and cervical cancer, from four eligible studies (28, 45, 49, 86) with 38,370 cases and 3,658,516 participants, specifically with an increase of CDDD to a high level, like 25,550 CDDD (OR:0.91; 95% CI: 0.85, 0.98, p linearity < 0.05, **Supplementary Material Figure 1**), compared to never AD users.

### Sensitivity analysis and publication bias

**Figure 5:**
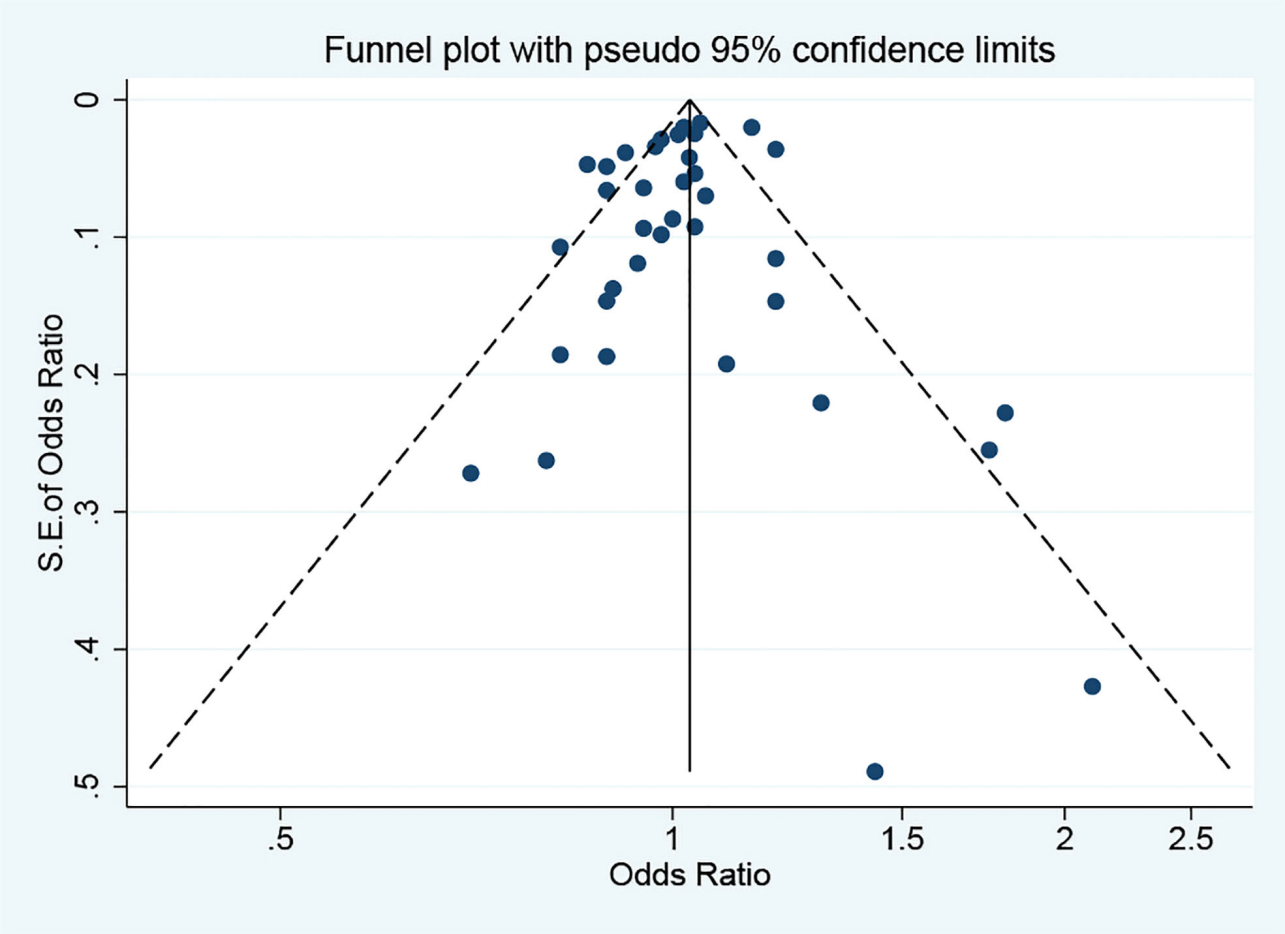
Funnel plot for identifying the publication bias. S.E., standard error. The circles alone are real studies. The vertical lines represent the summary effect estimates, and the dashed lines represent pseudo-95% confidence interval limits.

Applying the leave-one-out sensitivity analysis showed that none of the eligible studies had considerable effect on the overall estimate (**Supplementary Material Figure 2**). No publication bias was observed based on funnel plot symmetry (**Figure 5**) and results of Egger’s test (*p* = 0.354).

## Discussion

The pooled results of this systematic review and meta-analysis found that AD use did not increase the incidence risk of female breast and gynecological cancer, either by tricyclic ADs (pooled OR: 1; 95% CI: 0.97, 1.03) or by selective serotonin reuptake inhibitors (pooled OR: 0.99; 95% CI: 0.95, 1.04), and even decreased the incidence risk of ovarian cancer. Further subgroup analyses of confounders found that AD use was associated with higher risk in almost all of subgroups of unadjusted confounders; thus, the incomplete adjustment for these potential confounders was likely to bias our results toward a positive relationship. Additionally, further subgroup analyses of study characteristics found that the incidence risk of female breast and gynecological cancer was not statistically different across great majority strata, but it appeared to be stronger in exposures that occurred less than 1 year prior to the index date (1.25 for less than 1 year vs. 0.89 for more than 1 year) for ovarian cancer, as protopathic bias and reverse causation might drive the estimates towards an increased risk (88). It was also stronger in questionnaire for exposure assessment (1.02 for questionnaire vs. 0.89 for database) for ovarian cancer due to recall bias and selection bias by questionnaire for collecting information (88).

However, several potential hypotheses have been proposed regarding the biological mechanism underlying this positive association and have been supported by subsequent epidemiologic studies. However, most of them are limited for some reason, such as exposure misclassifications (27, 43), reverse causation bias (27, 37–40, 42, 43), recall bias, selection bias (27, 38–41), and unadjusted confounders (27, 42, 43). Moreover, the weight of epidemiologic evidence does not support the hypothesis that AD use increases the overall risk of breast and gynecological cancer (28, 44–63), which are consistent with our finding. Although the incidence risk of ovarian cancer is stronger in genotoxic TCA (pooled OR: 1.38; 95% CI: 0.78, 2.45) than that in nongenotoxic TCA, and in dopamine/norepinephrine reuptake inhibitors (pooled OR: 1.38; 95% CI: 0.79, 2.42) than that in serotonin alone or mixed with norepinephrine reuptake inhibitors in our finding, the results are only based on one (44) or three studies (36, 46, 50). Nevertheless, exposure to either genotoxic or nongenotoxic TCAs is not associated with a significant increase in the incidence of female breast cancer. In addition, Ashbury et al. (48) grouped SSRIs as higher and lower inhibitors dependent on the dissociation constant (*K*_d_) in order to accurately assess for levels of prolactin secreted by the secondary pituitary gland, and found that neither higher nor lower inhibitor of serotonin reuptake increased the risk for breast cancer, which was consistent with our result.

If AD medications work through changes in the secretion of gonadotropins and female sex hormones, the observed association may be more pronounced in premenopausal women who have functioning ovaries than that in postmenopausal women. Our results in this study support this hypothesis, but they are only based on limited two studies (40, 44) with possible bias for ovarian cancer. Conversely, prior prospective studies have linked prolactin with increased postmenopausal women breast cancer risk (89), but our results in this comprehensive study shows that these associations are weak or null as a whole. Moreover, different from several studies that have found that prolactin may encourage the development of estrogen receptor (ER)-positive tumors (89) and higher risk in in situ (52, 85), our study shows reverse results and/or weak associations, but they are based on limited studies and possible bias, respectively. Additionally, previous studies have shown a significant relationship between depression and the risk of cancer incidence (90). Our subgroup analyses in this study have also found that after adjusting the depressive symptoms, OR tends to slightly decrease for female breast cancer. It cannot be ruled out that depression itself may have an impact on cancer incidence. Thus, it is possible that the positive association observed between AD use and female breast cancer risk is due to depressive symptoms rather than AD use itself. Therefore, further studies are needed to figure it out.

Our stratified analyses for specific types of ADs indicated that the results were more pronounced in serotonin and norepinephrine reuptake inhibitors (SNRIs) for female breast, ovarian, endometrial, and cervical cancer, and stronger in noradrenergic and specific serotonergic antidepressants (NaSSas) for ovarian cancer. We also found a reduced risk of female breast cancer with the use of monoamine oxidase inhibitors (MAOIs) and serotonin antagonist and reuptake inhibitors (SARIs), and a reduced risk of endometrial, corpus uteri, and cervical cancer with the use of norepinephrine and dopamine reuptake inhibitors (NDRIs) and NaSSas. Amerio et al. reviewed the US Food and Drug Administration (FDA) preclinical *in vivo* evidence and found that 63.6% (7/11) of examined ADs were associated with carcinogenicity, including duloxetine (SNRIs), mirtazapine (NaSSas), and NDRI (bupropion), but the agents unassociated with carcinogenicity were trazodone (SARIs) and venlafaxine (SNRIs) (11). Meanwhile, several previous studies (91, 92) had reported the mechanisms on how mirtazapine (NaSSas) and MAOIs acted to inhibit tumor growth by enhancing immune function and causing neurotoxicity and repressing BHC110/LSD1, respectively. Conversely, a previous study has provided preliminary data of the possible association of trazodone (SARIs) and invasive cervical cancer (28). However, information pertaining to breast, uterine, and ovarian carcinogenesis clearly highlights that cervical cancer carcinogenesis is very different to the others, and is almost exclusively HPV driven and vastly different to that of the other organs analyzed, which is supported by the results of epidemiologic study that TCAs, MAOIs, and SSRIs are not associated with increased risk of invasive cervical cancer by Chan et al. (28). Furthermore, our results are based on limited one or two studies for each cancer site and short of adjusting the HPV infection as the critical potential confounders (28), and the incomplete adjustment for this potential confounder is likely to bias final results in an epidemiologic study. Thus, the above results are based on preliminary analysis and has so far proved inconclusive. Further large-scale prospective cohort studies adjusting for all possible confounding factors (including HPV infection) or animal and *in vitro* studies are needed to clarify the tumor-inhibiting or growth-promoting effect by different types of ADs and the biological mechanism underlying this association.

Furthermore, there is a non-linear dose–response relationship between the duration of AD use and incidence risk of female breast cancer, in which the bi-phasic phenomenon is characterized by “low-dose stimulation and high-dose inhibition” (12, 93) of malignant cell proliferation. This bi-phasic phenomenon shows that short-term use and/or low-dose AD may increase the risk of breast cancer in women or exacerbate cancer cell growth in women in the early stages of breast cancer, and long-term use and/or high dose inhibit tumor growth, which may help explain the high between-study heterogeneity for female breast cancer based on distinct dose or duration from different studies. Nevertheless, there is a positive linearity association between the CDDD of AD use and incidence risk of female breast cancer based on limited studies with possible bias (86). Additionally, an inverse linear dose–response association exists between the CDDD or duration of AD use and incidence risk of gynecological cancer, as well as between the CDDD and the incidence risk of endometrial, corpus uteri, and cervical cancer. Furthermore, the antiproliferative effects of AD use have been supported by previous studies (34, 44, 45) with a possible biological mechanism (31–33). However, this negative linearity association does not exist in individual cancer sites partly due to the limited numbers of studies and the strong non-linearity or linearity phenomenon that only happens with an increase of CDDD or duration to a high level. Thus, further large-scale prospective cohort studies specifying dose or duration are needed to accurately assess and clarify the protective or bi-phasic effect and biological mechanism.

### Strength and limitation

Notably, our meta-analysis has the following advantages. First, to the best of our knowledge, this is the first study to systematically perform a qualitative dose–response meta-analysis of the relationship between AD use and the incidence of female breast and gynecological cancer as a whole of hormone-related cancer in female patients, along with comprehensive subgroup analyses stratified by almost all study characteristics and important potential confounders. Furthermore, this is the first study to demonstrate shapes of non-linear or linear association between the CDDD or duration of AD use and female breast or gynecological cancer, and further clarify and validate the several important potential hypotheses regarding the biological mechanism underlying this association.

Inevitably, this study also has some limitations. First, moderate or high heterogeneity among studies was observed when pooling estimates for female breast cancer. Furthermore, a small portion of subgroup analyses of study characteristics and important potential confounders was based on a limited number of existing studies. Second, a linearity association was not observed in the relation between the CDDD or duration of AD use and the incidence risk of ovarian, endometrial, corpus uteri, and cervical cancer, respectively, partly due to the limited number of existing studies for each cancer site. Finally, the interpretation criteria for exposure were inconsistent, and misclassification bias might affect the results.

## Conclusion

Overall, the results of the present updated meta-analysis involving the largest sample size to date and mostly included comprehensive observational studies show that AD use does not increase the incidence risk of female breast and gynecological cancer, either by TCA or by SSRI, and even decreases the incidence risk of ovarian cancer, compared to never AD users. There is a non-linear dose–response relationship between the duration of AD use and incidence risk of female breast cancers, with a very slight increase in incidence risk of female breast cancer on short-term usage. An inverse linearity association exists between the CDDD or duration of AD use and incidence risk of gynecological cancer, and also between the CDDD of AD use and incidence risk of endometrial, corpus uteri, and cervical cancer. More future studies specifying dose or duration are needed in order to accurately assess and clarify this protective or bi-phasic effect and biological mechanism.

## Data availability statement

The original contributions presented in the study are included in the article/**Supplementary Material**. Further inquiries can be directed to the corresponding authors.

## Author contributions

HS, YYL, and YZ conceived and designed the study. HS, YYL, and YZ selected the studies and collected the data. YYL and YZ analyzed data. All authors interpreted the results. YYL and YZ drafted the paper. All authors revised the draft paper. All authors contributed to the article and approved the submitted version.

## Funding

This study was supported by the Joint Funds for the National Key Research and Development Program of China (Grant No. 2021ZD0202900), the National Natural Science Foundation of China (Grant Nos. 62027812, 81771470, and 82101608), and the Natural Science Foundation of Shandong Province (Grant No. ZR2020ZD17). The funders of this study had no role in study design, collection, analysis, interpretation of data, the writing of this article, or the decision to submit it for publication. The corresponding authors had full access to all the data in the study and had final responsibility for the decision to submit for publication.

## Conflict of interest

The authors declare that the research was conducted in the absence of any commercial or financial relationships that could be construed as a potential conflict of interest.

## Publisher’s note

All claims expressed in this article are solely those of the authors and do not necessarily represent those of their affiliated organizations, or those of the publisher, the editors and the reviewers. Any product that may be evaluated in this article, or claim that may be made by its manufacturer, is not guaranteed or endorsed by the publisher.
