# Corrigendum: The incidence risk of breast and gynecological cancer by antidepressant use: A systematic review and dose–response meta-analysis of epidemiological studies involving 160,727 patients

**DOI:** 10.3389/fonc.2022.1072594

**Published:** 2022-11-08

**Authors:** Yanjia Zhuang, Xiaogang Pang, Yuchen Qi, Tianshu Zhang, Guimao Cao, Heming Xue, Yifan Xu, Shuoxin Xie, Yifan Liu, Yinuo Wang, Yunxiao Li, Ying Xiong, Yuanyuan Li, Hui Shen

**Affiliations:** ^1^Laboratory of Brain Science, Innovation Research Institute of Traditional Chinese Medicine, Shandong University of Traditional Chinese Medicine, Jinan, China; ^2^Experimental Center, Shandong University of Traditional Chinese Medicine, Jinan, China; ^3^School of health, Shandong University of Traditional Chinese Medicine, Jinan, China; ^4^Department of Anesthesiology, Affiliated Hospital of Shandong University of Traditional Chinese Medicine, Jinan, China; ^5^School of Medicine, Shandong University of Traditional Chinese Medicine, Jinan, China; ^6^School of Acupuncture, Shandong University of Traditional Chinese Medicine, Jinan, China; ^7^Innovation Research Institute of Traditional Chinese Medicine, Shandong University of Traditional Chinese Medicine, Jinan, China

**Keywords:** antidepressant, depression, breast cancer, gynecological cancer, Incidence, meta-analysis, systematic review, dose-response analysis

In the published article, there were three instances where there was a mistake in the cited references. This is because the references were renumbered sequentially after revision of manuscript.

In the Results section, the sentence “By comparison, a non-linear dose–response meta-analysis with 13 eligible studies (37-39, 42, 48, 55-57, 60, 61, 66, 83, 84)” has been changed to “By comparison, a non-linear dose–response meta-analysis with 13 eligible studies (37, 38, 42, 47, 48, 55-57, 60, 61, 66, 83, 84)”.

In the Results section, the sentence “Another negative linearity association also existed between the CDDD and the incidence risk of endometrial, corpus uteri, and cervical cancer, from four eligible studies (30, 33, 34, 72)” has been changed to “Another negative linearity association also existed between the CDDD and the incidence risk of endometrial, corpus uteri, and cervical cancer, from four eligible studies (28, 45, 49, 86)”.

In the Discussion section, the sentence “Moreover, different from several studies that have found that prolactin may encourage the development of estrogen receptor (ER)-positive tumors (89) and higher risk in in situ (51, 85)” has been changed to “Moreover, different from several studies that have found that prolactin may encourage the development of estrogen receptor (ER)-positive tumors (89) and higher risk in in situ (52, 85)”.

The authors apologize for these errors and state that this does not change the scientific conclusions of the article in any way. The original article has been updated.

## Publisher’s note

All claims expressed in this article are solely those of the authors and do not necessarily represent those of their affiliated organizations, or those of the publisher, the editors and the reviewers. Any product that may be evaluated in this article, or claim that may be made by its manufacturer, is not guaranteed or endorsed by the publisher.

