# Supplementary material for: The incidence risk of breast and gynecological cancer by antidepressant use: A systematic review and dose–response meta-analysis of epidemiological studies involving 160,727 patients
Source: Front Oncol. 2022 Oct 14;12:939636. doi: 10.3389/fonc.2022.939636 (PMC9680975; doi:10.3389/fonc.2022.939636)

frontiers

# *Supplementary Material*

**Supplementary Table 1. MOOSE checklist**

*on the supplementary materials

| Item No | Recommendation | Reported on Page No |
|---|---|---|
| Reporting of background should include | | |
| 1 | Problem definition | 2 |
| 2 | Hypothesis statement | 3 |
| 3 | Description of study outcome(s) | 3,4 |
| 4 | Type of exposure or intervention used | 3 |
| 5 | Type of study designs used | 3 |
| 6 | Study population | 3 |
| Reporting of search strategy should include | | |
| 7 | Qualifications of searchers (eg, librarians and investigators) | 3,4 |
| 8 | Search strategy, including time period included in the synthesis and key words | 3,Table 2* |
| 9 | Effort to include all available studies, including contact with authors | 3, without contact authors |
| 10 | Databases and registries searched | 3 |
| 11 | Search software used, name and version, including special features used (eg, explosion) | 3 |
| 12 | Use of hand searching (eg, reference lists of obtained articles) | NA |
| 13 | List of citations located and those excluded, including justification | Figure 1, Table 4* |
| 14 | Method of addressing articles published in languages other than English | NA |
| 15 | Method of handling abstracts and unpublished studies | NA |
| 16 | Description of any contact with authors | NA |
| Reporting of methods should include | | |
| 17 | Description of relevance or appropriateness of studies assembled for | 3 |

| | | |
|---|---|---|
| | assessing the hypothesis to be tested | |
| 18 | Rationale for the selection and coding of data (eg, sound clinical principles or convenience) | 3,4 |
| 19 | Documentation of how data were classified and coded (eg, multiple raters, blinding and interrater reliability) | 3,4 |
| 20 | Assessment of confounding (eg, comparability of cases and controls in studies where appropriate) | 4-6 |
| 21 | Assessment of study quality, including blinding of quality assessors, stratification or regression on possible predictors of study results | 5 |
| 22 | Assessment of heterogeneity | 4-6 |
| 23 | Description of statistical methods (eg, complete description of fixed or random effects models, justification of whether the chosen models account for predictors of study results, dose-response models, or cumulative meta-analysis) in sufficient detail to be replicated | 4 |
| 24 | Provision of appropriate tables and graphics | Figure 1-5<br>Table 1-2<br>Table1-4*<br>Figure1-2* |
| Reporting of results should include | | |
| 25 | Table giving descriptive information for each study included | Table 1 |
| 26 | Results of sensitivity testing (eg, subgroup analysis) | Table 2, Figure 2* |
| 27 | Indication of statistical uncertainty of findings | 6-8 |
| Reporting of Discussion | | |
| 28 | Quantitative assessment of bias (eg, publication bias) | Figure 5 |
| 29 | Justification for exclusion (eg, exclusion of non–English-language citations) | Figure 1, Table 4* |
| 30 | Assessment of quality of included studies | Table 3* |
| Reporting of Conclusions | | |
| 31 | Consideration of alternative explanations for observed results | 6-8 |
| 32 | Generalization of the conclusions (ie, appropriate for the data presented and within the domain of the literature review) | 8 |
| 33 | Guidelines for future research | 8 |
| 34 | Disclosure of funding source | 8-9 |

From:  Brooke BS, Schwartz TA, Pawlik TM. MOOSE Reporting Guidelines for Meta-analyses of Observational Studies. *JAMA Surg.* 2021;156(8):787–788. doi:10.1001/jamasurg.2021.0522

**Supplementary Table 2. Details of search strategy**

| **Database** | **PubMed.** |
|---|---|
| **Search Terms** | #1 "Neoplasms"[Mesh]<br><br>#2 cancer*[Text word] OR Tumor*[Text word] OR malignan*[Text word] OR neoplas*[Text word] OR tumour*[Text word] OR carcinoma*[Text word] OR adenocarcinoma*[Text word] OR choriocarcinoma*[Text word] OR lymphoma*[Text word] OR leukemia*[Text word] OR leukaemia*[Text word] OR metastat*[Text word] OR sarcoma*[Text word] OR teratoma*[Text word] OR Epithelioma*[Text word]<br><br>#3 #1 OR #2<br><br>#4 "Antidepressive Agents"[MeSH Terms] OR "Serotonin Uptake Inhibitors"[MeSH Terms] OR "Adrenergic Uptake Inhibitors"[MeSH Terms] OR "Monoamine Oxidase Inhibitors"[MeSH Terms]<br><br>#5 "anti-depress*"[Text Word] OR "trycyclic*"[Text Word] OR "tca"[Text Word] OR "ssri*"[Text Word] OR "serotonin uptake*"[Text Word] OR "snri*"[Text Word] OR "monoamine oxidase inhibitor*"[Text Word] OR "maoi*"[Text Word] OR "mao inhibitor*"[Text Word] OR "thymoanaleptic*"[Text Word] OR "thymoleptic*"[Text Word] OR "5 hydroxytryptamine*"[Text Word] OR "5 ht*"[Text Word] OR "rima*"[Text Word]<br><br>#6 "desipramine*"[Text Word] OR "imipramine*"[Text Word] OR "clomipramine*"[Text Word] OR "opipramol*"[Text Word] OR "trimipramine*"[Text Word] OR "lofepramine*"[Text Word] OR "dibenzepin*"[Text Word] OR "amitriptyline*"[Text Word] OR "nortriptyline*"[Text Word] OR "protriptyline*"[Text Word] OR "doxepin*"[Text Word] OR "iprindole*"[Text Word] OR "melitracen*"[Text Word] OR "butriptyline*"[Text Word] OR "dosulepin*"[Text Word] OR "amoxapine*"[Text Word] OR "dimetacrine*"[Text Word] OR "amineptine*"[Text Word] OR "maprotiline*"[Text Word] OR "quinupramine*"[Text Word] OR "zimeldine*"[Text Word] OR "fluoxetine*"[Text Word] OR "citalopram*"[Text Word] OR "paroxetine*"[Text Word] OR "sertraline*"[Text Word] OR "alaproclate*"[Text Word] OR "fluvoxamine*"[Text Word] OR "etoperidone*"[Text Word] OR "escitalopram*"[Text Word] OR "isocarboxazid*"[Text Word] OR "nialamide*"[Text Word] OR "phenelzine*"[Text Word] OR "tranylcypromine*"[Text Word] OR "iproniazide*"[Text Word] OR "iproclozide*"[Text Word] OR "moclobemide*"[Text Word] OR "toloxatone*"[Text Word] OR "oxitriptan*"[Text Word] OR "tryptophan*"[Text Word] OR "mianserin*"[Text Word] OR "nomifensine*"[Text Word] OR "trazodone*"[Text Word] OR "nefazodone*"[Text Word] OR "minaprine*"[Text Word] OR "bifemelane*"[Text Word] OR "viloxazine*"[Text Word] OR "oxaflozane*"[Text Word] OR |

| | |
|---|---|
| | "mirtazapine*"[Text Word] OR "bupropion*"[Text Word] OR "medifoxamine*"[Text Word] OR "tianeptine*"[Text Word] OR "pivagabine*"[Text Word] OR "venlafaxine*"[Text Word] OR "milnacipran*"[Text Word] OR "reboxetine*"[Text Word] OR "gepirone*"[Text Word] OR "duloxetine*"[Text Word] OR "agomelatine*"[Text Word] OR "desvenlafaxine*"[Text Word] OR "vilazodone*"[Text Word] OR "hyperici herba*"[Text Word] OR "hypericum perforatum*"[Text Word] OR "st john* wort*"[Text Word] OR "vortioxetine*"[Text Word] OR "esketamine*"[Text Word] OR "levomilnacipran*"[Text Word] OR "Clovoxamine*"[Text Word] OR "Clorgyline*"[Text Word] OR "Dothiepin*"[Text Word] OR "Sulpiride*"[Text Word] OR "Rolipram*"[Text Word]<br><br>#7 #4 OR #5 OR #6<br><br>#8 "morbidity"[MeSH Terms] OR "incidence"[MeSH Terms] OR "risk"[MeSH Terms]<br><br>#9 "morbidit*"[Text Word] OR "incidence*"[Text Word] OR "occurrence*"[Text Word] OR "genesis*"[Text Word] OR "occur*"[Text Word] OR "risk*"[Text Word]<br><br>#10 #8 OR #9<br><br>#11 "Case-Control Studies"[MeSH Terms] OR "Cohort Studies"[MeSH Terms]<br><br>#12 "case*"[Text Word] AND ("control*"[Text Word] OR "comparison*"[Text Word] OR "compeer*"[Text Word] OR "referrent*"[Text Word] OR "base*"[Text Word])<br><br>#13 "cohort*"[Text Word] OR "longitudinal*"[Text Word] OR "prospective*"[Text Word] OR "follow-up*"[Text Word] OR "retrospective*"[Text Word]<br><br>#14 #11 OR #12 OR #13<br><br>#15 #14 AND #10 AND #7 AND #3<br><br>#16 "Animals"[Mesh]<br><br>#17 "Humans"[Mesh]<br><br>#18 #16 NOT #17<br><br>#19 #15 NOT #18 |
| **Database** | **Web of Science** |
| **Search** | #1 TS=(Antidepressive Agent* or Serotonin Uptake Inhibitor* or Adrenergic Uptake Inhibitors* or Monoamine Oxidase Inhibitor* or anti-depress* or |

| | |
|---|---|
| **Terms** | antidepress* or trycyclic* or TCA* or serotonin uptake or SSRI* or SNRI* or monoamine oxidase inhibitor* or MAOI* or mao inhibitor* or thymoanaleptic* or thymoleptic* or 5 hydroxytryptamine* or 5-hydroxytryptamine* or 5 ht or 5-ht* or rima* or desipramine* or imipramine* or clomipramine* or opipramol* or trimipramine* or lofepramine* or dibenzepin* or amitriptyline* or nortriptyline* or protriptyline* or doxepin* or iprindole* or melitracen* or butriptyline* or dosulepin* or amoxapine* or dimetacrine* or amineptine* or maprotiline* or quinupramine* or zimeldine* or fluoxetine* or citalopram* or paroxetine* or sertraline* or alaproclate* or fluvoxamine* or etoperidone* or escitalopram* or isocarboxazid* or nialamide* or phenelzine* or tranylcypromine* or iproniazide* or iproclozide* or moclobemide* or toloxatone* or oxitriptan* or tryptophan* or mianserin* or nomifensine* or trazodone* or nefazodone* or minaprine* or bifemelane* or viloxazine* or oxaflozane* or mirtazapine* or bupropion* or medifoxamine* or tianeptine* or pivagabine* or venlafaxine* or milnacipran* or reboxetine* or gepirone* or duloxetine* or agomelatine* or desvenlafaxine* or vilazodone* or hyperici herba* or hypericum perforatum* or st john* wort* or vortioxetine* or esketamine* or levomilnacipran* or Clovoxamine* or Clorgyline* or Dothiepin* or Sulpiride* or Rolipram*)<br><br># 2 (TS=(case*)) AND TS=((control* or comparison* or compeer* or referrent* or base*))<br><br># 3 TS=(cohort* or longitudinal* or prospective* or retrospective* or follow-up*)<br><br># 4 #2 OR #3<br><br># 5 TS=(morbidit* or incidence* or occurrence* or genesis* or occur* or risk*)<br><br># 6 TS=(Neoplasms* or cancer* or tumor* or tumour* or neoplas* or malignan* or carcinoma* or adenocarcinoma* or choriocarcinoma* or lymphoma* or leukemia* or leukaemia* or metastat* or sarcoma* or teratoma* or epithelioma*)<br><br>#7 #1 AND #4 AND #5 AND #6 |
| **Database** | **Embase** |
| **Search Terms** | #1 'neoplasm'/exp OR cancer*:ti,ab,kw OR tumor*:ti,ab,kw OR malignan*:ti,ab,kw OR neoplas*:ti,ab,kw OR tumour*:ti,ab,kw OR carcinoma*:ti,ab,kw OR adenocarcinoma*:ti,ab,kw OR choriocarcinoma*:ti,ab,kw OR lymphoma*:ti,ab,kw OR leukemia*:ti,ab,kw OR leukaemia*:ti,ab,kw OR metastat*:ti,ab,kw OR sarcoma*:ti,ab,kw OR teratoma*:ti,ab,kw OR epithelioma*:ti,ab,kw<br><br>#2 desipramine*:ti,ab,kw OR imipramine*:ti,ab,kw OR clomipramine*:ti,ab,kw OR opipramol*:ti,ab,kw OR trimipramine*:ti,ab,kw OR lofepramine*:ti,ab,kw OR dibenzepin*:ti,ab,kw OR amitriptyline*:ti,ab,kw OR nortriptyline*:ti,ab,kw OR protriptyline*:ti,ab,kw OR doxepin*:ti,ab,kw OR iprindole*:ti,ab,kw OR |

| | |
|---|---|
| | melitracen*:ti,ab,kw OR butriptyline*:ti,ab,kw OR dosulepin*:ti,ab,kw OR amoxapine*:ti,ab,kw OR dimetacrine*:ti,ab,kw OR amineptine*:ti,ab,kw OR maprotiline*:ti,ab,kw OR quinupramine*:ti,ab,kw OR zimeldine*:ti,ab,kw OR fluoxetine*:ti,ab,kw OR citalopram*:ti,ab,kw OR paroxetine*:ti,ab,kw OR sertraline*:ti,ab,kw OR alaproclate*:ti,ab,kw OR fluvoxamine*:ti,ab,kw OR etoperidone*:ti,ab,kw OR escitalopram*:ti,ab,kw OR isocarboxazid*:ti,ab,kw OR nialamide*:ti,ab,kw OR phenelzine*:ti,ab,kw OR tranylcypromine*:ti,ab,kw OR iproniazide*:ti,ab,kw OR iproclozide*:ti,ab,kw OR moclobemide*:ti,ab,kw OR toloxatone*:ti,ab,kw OR oxitriptan*:ti,ab,kw OR tryptophan*:ti,ab,kw OR mianserin*:ti,ab,kw OR nomifensine*:ti,ab,kw OR trazodone*:ti,ab,kw OR nefazodone*:ti,ab,kw OR minaprine*:ti,ab,kw OR bifemelane*:ti,ab,kw OR viloxazine*:ti,ab,kw OR oxaflozane*:ti,ab,kw OR mirtazapine*:ti,ab,kw OR bupropion*:ti,ab,kw OR medifoxamine*:ti,ab,kw OR tianeptine*:ti,ab,kw OR pivagabine*:ti,ab,kw OR venlafaxine*:ti,ab,kw OR milnacipran*:ti,ab,kw OR reboxetine*:ti,ab,kw OR gepirone*:ti,ab,kw OR duloxetine*:ti,ab,kw OR agomelatine*:ti,ab,kw OR desvenlafaxine*:ti,ab,kw OR vilazodone*:ti,ab,kw OR 'hyperici herba*':ti,ab,kw OR 'hypericum perforatum*':ti,ab,kw OR 'st john* wort*':ti,ab,kw OR vortioxetine*:ti,ab,kw OR esketamine*:ti,ab,kw OR levomilnacipran*:ti,ab,kw OR clovoxamine*:ti,ab,kw OR clorgyline*:ti,ab,kw OR sulpiride*:ti,ab,kw OR dothiepin*:ti,ab,kw OR rolipram*:ti,ab,kw<br><br>#3 'morbidity'/exp OR 'incidence'/exp OR 'risk'/exp<br><br>#4 morbidit*:ti,ab,kw OR incidence*:ti,ab,kw OR occurrence*:ti,ab,kw OR genesis*:ti,ab,kw OR occur*:ti,ab,kw OR risk*:ti,ab,kw<br><br>#5 #3 OR #4<br><br>#6 'case control study'/exp OR 'cohort analysis'/exp<br><br>#7 control*:ti,ab,kw OR comparison*:ti,ab,kw OR compeer*:ti,ab,kw OR referrent*:ti,ab,kw OR base*:ti,ab,kw<br><br>#8 cohort*:ti,ab,kw OR longitudinal*:ti,ab,kw OR prospective*:ti,ab,kw OR 'follow up*':ti,ab,kw OR retrospective*:ti,ab,kw<br><br>#9 case*:ti,ab,kw<br><br>#10 #7 AND #9<br><br>#11 #6 OR #8 OR #10<br><br>#12 antidepressent*:ti,ab,kw OR 'anti-depress*':ti,ab,kw OR trycyclic*:ti,ab,kw OR tca*:ti,ab,kw OR ssri*:ti,ab,kw OR 'serotonin uptake*':ti,ab,kw OR snri*:ti,ab,kw OR maoi*:ti,ab,kw OR 'mao inhibitor*':ti,ab,kw OR thymoanaleptic*:ti,ab,kw OR thymoleptic*:ti,ab,kw OR '5 hydroxytryptamine*':ti,ab,kw OR rima*:ti,ab,kw OR 'monoamine oxidase |

| | |
|---|---|
| | inhibitor*':ti,ab,kw OR '5 ht*':ti,ab,kw<br><br>#13 'antidepressant agent'/exp<br><br>#14 #2 OR #12 OR #13<br><br>#15 #1 AND #5 AND #11 AND #14<br><br>#16 #15 AND 'human'/de |
| **Database** | **The Cochrane Library** |
| **Search Terms** | #1 MeSH descriptor: [Neoplasms] explode all trees<br><br>#2 (cancer* or tumor* or tumour* or neoplas* or malignan* or carcinoma* or adenocarcinoma* or choriocarcinoma* or lymphoma* or leukemia* or leukaemia* or metastat* or sarcoma* or teratoma* or epithelioma*)<br><br>#3 #1 or #2<br><br>#4 MeSH descriptor: [Antidepressive Agents] explode all trees<br><br>#5 MeSH descriptor: [Serotonin Uptake Inhibitors] explode all trees<br><br>#6 MeSH descriptor: [Adrenergic Uptake Inhibitors] explode all trees<br><br>#7 MeSH descriptor: [Monoamine Oxidase Inhibitors] explode all trees<br><br>#8 MeSH descriptor: [Psychotropic Drugs] explode all trees<br><br>#9 (psychotropic drug* or anti-depress* or antidepress* or trycyclic* or TCA* or serotonin uptake or SSRI* or SNRI* or monoamine oxidase inhibitor* or MAOI* or mao inhibitor* or thymoanaleptic* or thymoleptic* or 5 hydroxytryptamine* or 5 ht or rima*)<br><br>#13 #4 or #5 or #6 or #7 or #8 or #9 or #11<br><br>#14 MeSH descriptor: [Morbidity] explode all trees<br><br>#15 MeSH descriptor: [Incidence] explode all trees<br><br>#16 MeSH descriptor: [Risk] explode all trees<br><br>#17 (morbidit* or incidence* or occurrence* or genesis* or occur* or risk*) |

| | |
|---|---|
| | #18 #14 or #15 or #16 or #17<br><br>#19 MeSH descriptor: [Case-Control Studies] explode all trees<br><br>#20 MeSH descriptor: [Cohort Studies] explode all trees<br><br>#21 (cohort* or longitudinal* or prospective* or retrospective* or follow-up*)<br><br>#22 case*<br><br>#23 (control* or comparison* or compeer* or referent* or base*)<br><br>#24 #22 and #23<br><br>#25 #24 or #19 or #20 or #21<br><br>#27 #25 and #18 and #13 and #3 |
| **Database** | **PsycINFO** |
| **Search Terms** | #1 exp Neoplasms/<br><br>#2 (cancer* or tumor* or tumour* or neoplas* or malignan* or carcinoma* or adenocarcinoma* or choriocarcinoma* or lymphoma* or leukemia* or leukaemia* or metastat* or sarcoma* or teratoma* or epithelioma*).mp.<br><br>#3 1 or 2<br><br>#4 exp Monoamine Oxidase Inhibitors/<br><br>#5 exp serotonin reuptake inhibitors/<br><br>#6 exp antidepress/<br><br>#7 exp Psychotropic Drugs/<br><br>#8 (psychotropic drug* or anti-depress* or antidepress* or trycyclic* or TCA* or serotonin uptake or SSRI* or SNRI* or monoamine oxidase inhibitor* or MAOI* or mao inhibitor* or thymoanaleptic* or thymoleptic* or 5 hydroxytryptamine* or 5 ht or rima*).mp.<br><br>#9 (anti-depress* or antidepress* or trycyclic* or TCA* or serotonin uptake or SSRI* or SNRI* or monoamine oxidase inhibitor* or MAOI* or mao inhibitor* or thymoanaleptic* or thymoleptic* or 5 hydroxytryptamine* or 5 ht or rima*).mp. |

| | |
|---|---|
| | #10 (desipramine* or imipramine* or clomipramine* or opipramol* or trimipramine* or lofepramine* or dibenzepin* or amitriptyline* or nortriptyline* or protriptyline* or doxepin* or iprindole* or melitracen* or butriptyline* or dosulepin* or amoxapine* or dimetacrine* or amineptine* or maprotiline* or quinupramine* or zimeldine* or fluoxetine* or citalopram* or paroxetine* or sertraline* or alaproclate* or fluvoxamine* or etoperidone* or escitalopram* or isocarboxazid* or nialamide* or phenelzine* or tranylcypromine* or iproniazide* or iproclozide* or moclobemide* or toloxatone* or oxitriptan* or tryptophan* or mianserin* or nomifensine* or trazodone* or nefazodone* or minaprine* or bifemelane* or viloxazine* or oxaflozane* or mirtazapine* or bupropion* or medifoxamine* or tianeptine* or pivagabine* or venlafaxine* or milnacipran* or reboxetine* or gepirone* or duloxetine* or agomelatine* or desvenlafaxine* or vilazodone* or hyperici herba* or hypericum perforatum* or st john* wort* or vortioxetine* or esketamine* or levomilnacipran* or Clovoxamine* or Clorgyline* or Dothiepin* or Sulpiride* or Rolipram*).mp.<br><br>#11 (morbidit* or incidence* or occurrence* or genesis* or occur* or risk*).mp.<br><br>#12 exp Morbidity/<br><br>#13 11 or 12<br><br>#14 exp Cohort analysis/<br><br>#15 exp Longitudinal Studies/<br><br>#16 exp Prospective Studies/<br><br>#17 (cohort* or longitudinal* or prospective* or retrospective* or follow-up*).mp.<br><br>#18 case*.mp.<br><br>#19 (control* or comparison* or compeer* or referrent* or base*).mp.<br><br>#20 18 and 19<br><br>#21 14 or 15 or 16 or 17 or 20<br><br>#22 4 or 5 or 6 or 7 or 8 or 10<br><br>#23 3 and 13 and 21 and 22<br><br>#24 4 or 5 or 6 or 9 or 10<br><br>#25 3 and 13 and 21 and 24 |

Supplementary Table 3. Newcastle-Ottawa quality scale for study assessment

| Case–control study (year, cancer types) | Selection | | | | Control for important factor or additional factor | Exposure | | | Total quality scores |
|---|---|---|---|---|---|---|---|---|---|
| | Adequate definition of cases | Representa tiveness of cases | Selection of control subjects | Definition of control subjects | | Exposure assessment | Same method of ascertainment for all subjects | Non response Rate | |
| Sperling et al. (2021, endometrial) | 1 | 1 | 1 | 1 | 2 | 1 | 1 | 0 | 8 |
| Busby et al. (2018, breast) | 1 | 1 | 0 | 1 | 2 | 1 | 1 | 0 | 7 |
| Morch et al. (2017, ovarian) | 1 | 1 | 1 | 0 | 2 | 1 | 1 | 0 | 7 |
| Lin et al. (2016, endometrial) | 1 | 1 | 1 | 1 | 1 | 1 | 1 | 0 | 7 |
| Boursi et al. (2015, breast) | 1 | 1 | 1 | 1 | 1 | 1 | 1 | 0 | 7 |

| | | | | | | | | | |
|---|---|---|---|---|---|---|---|---|---|
| Chan et al. (2015, cervical) | 1 | 1 | 1 | 1 | 1 | 1 | 1 | 0 | 7 |
| Wu et al. (2015, ovarian) | 1 | 1 | 1 | 1 | 1 | 1 | 1 | 0 | 7 |
| Ashbury et al. (2012, breast) | 1 | 1 | 1 | 0 | 1 | 1 | 1 | 0 | 6 |
| Walker et al. (2011, breast) | 1 | 1 | 1 | 1 | 1 | 1 | 1 | 0 | 7 |
| Wernli et al. (2009, breast) | 1 | 1 | 1 | 0 | 2 | 0 | 1 | 0 | 6 |
| Coogan et al. (2008, breast) | 1 | 1 | 0 | 1 | 2 | 0 | 1 | 0 | 6 |
| Davis et al. (2007,breast) | 1 | 1 | 1 | 0 | 2 | 0 | 1 | 0 | 6 |
| Chien et al. (2006,breast) | 1 | 1 | 1 | 1 | 2 | 0 | 1 | 0 | 7 |

| | | | | | | | | | |
|---|---|---|---|---|---|---|---|---|---|
| Fulton-Kehoe et al. (2006,breast) | 1 | 1 | 1 | 0 | 2 | 1 | 1 | 0 | 7 |
| Tamin et al. (2006, breast) | 1 | 1 | 1 | 1 | 1 | 1 | 1 | 0 | 7 |
| Coogan et al. (2005, breast) | 1 | 1 | 0 | 1 | 1 | 0 | 1 | 0 | 5 |
| Gonzalez-Perez et al. (2005, breast) | 1 | 1 | 1 | 0 | 1 | 1 | 1 | 0 | 6 |
| Moorman et al. (2005, ovarian) | 1 | 1 | 1 | 1 | 1 | 0 | 1 | 0 | 6 |
| Moorman et al. (2003, breast) | 1 | 1 | 1 | 0 | 2 | 0 | 1 | 0 | 6 |
| Steingart et al. (2003, breast) | 1 | 1 | 1 | 1 | 2 | 0 | 1 | 0 | 7 |
| Dublin et al. (2002, ovarian) | 1 | 1 | 1 | 0 | 1 | 1 | 1 | 0 | 6 |

| | | | | | | | | | |
|---|---|---|---|---|---|---|---|---|---|
| Sharpe et al. (2002, breast) | 1 | 1 | 1 | 0 | 0 | 1 | 1 | 0 | 5 |
| Cotterchio et al. (2000, breast) | 1 | 1 | 1 | 0 | 1 | 0 | 1 | 0 | 5 |
| Coogan et al. (2000, ovarian) | 1 | 1 | 0 | 1 | 1 | 0 | 1 | 0 | 5 |
| Kelly et al. (1999, breast) | 1 | 1 | 0 | 1 | 2 | 0 | 1 | 0 | 6 |
| Harlow et al. (1998, ovarian) | 1 | 1 | 1 | 0 | 2 | 0 | 1 | 0 | 6 |
| Harlow et al. (1995, ovarian) | 1 | 1 | 1 | 0 | 2 | 0 | 1 | 0 | 6 |

| **Cohort study (year, cancer types)** | **Selection** | | | | **Comparability of cohorts on the basis of the design or analysis** | **Outcome** | | | **Total quality scores** |
|---|---|---|---|---|---|---|---|---|---|
| | **Representativeness of the** | **Selection of the** | **Ascertainment of** | **Demonstration that** | | **Assessment** | **Was follow-up long enough** | **Adequacy of** | |

| | exposed cohort | nonexposed cohort | exposure | outcome of interest was not present at start of study | | of outcome | for outcomes to occur? | follow-up of cohort | |
|---|---|---|---|---|---|---|---|---|---|
| Reeves et al.(2018, breast) | 0 | 1 | 0 | 1 | 2 | 1 | 1 | 1 | 7 |
| Brown et al. (2016, breast) | 1 | 1 | 0 | 1 | 2 | 1 | 1 | 0 | 7 |
| Chen et al. (2016, breast) | 1 | 1 | 1 | 1 | 1 | 1 | 1 | 1 | 8 |
| Sun et al. (2015, breast) | 1 | 1 | 1 | 1 | 1 | 1 | 1 | 1 | 8 |
| Haukka et al. (2010, breast, ovarian ，Corpus uteri) | 1 | 1 | 1 | 1 | 0 | 1 | 1 | 0 | 6 |
| Wang et al. (2001, breast) | 1 | 1 | 1 | 1 | 1 | 1 | 1 | 0 | 7 |

| | | | | | | | | | |
|---|---|---|---|---|---|---|---|---|---|
| Kato et al. (2000, breast/ovarian/endometrial) | 1 | 1 | 0 | 1 | 1 | 1 | 1 | 1 | 7 |

**Supplementary Table 4. Reasons for Exclusion in Full-text Assessment**

| No. | Full text being excluded | Reasons |
|---|---|---|
| 1 | Dalton SO, Johansen C, Mellemkjaer L, et al. Antidepressant medications and risk for cancer. *Epidemiology*. 2000;11(2):171-176. doi:10.1097/00001648-200003000-00015 | shorting the data of incidence risk estimates of breast and gynecological cancer by antidepressant use |
| 2 | Shim EJ, Lee JW, Cho J, et al. Association of depression and anxiety disorder with the risk of mortality in breast cancer: A National Health Insurance Service study in Korea. *Breast Cancer Res Treat*. 2020;179(2):491-498. doi:10.1007/s10549-019-05479-3 | shorting the data of incidence risk estimates of breast and gynecological cancer by antidepressant use |
| 3 | Lokugamage AU, Hotopf M, Hardy R, et al. Breast cancer in relation to childhood parental divorce and early adult psychiatric disorder in a British birth cohort. *Psychol Med*. 2006;36(9):1307-1312. doi:10.1017/S0033291706007914 | shorting the data of incidence risk estimates of breast and gynecological cancer by antidepressant use |
| 4 | Liang X, Margolis KL, Hendryx M, et al. Effect of depression before breast cancer diagnosis on mortality among postmenopausal women. *Cancer*. 2017;123(16):3107-3115. doi:10.1002/cncr.30688 | shorting the data of incidence risk estimates of breast and gynecological cancer by antidepressant use |
| 5 | Haque R, Enger SM, Chen W, Petitti DB. Breast cancer risk in a large cohort of female antidepressant medication users. *Cancer Lett*. 2005;221(1):61-65. doi:10.1016/j.canlet.2004.11.003 | shorting the data of incidence risk estimates of breast and gynecological cancer by antidepressant use |
| 6 | Eskelinen M, Ollonen P. Forsen psychological risk inventory for breast cancer patients: a prospective case-control study with special reference to the use of psychiatric medications. *Anticancer Res*. 2011;31(2):739-744. | shorting the data of incidence risk estimates of breast and gynecological cancer by antidepressant use |
| 7 | Davis S, Mirick DK. Residential magnetic fields, medication use, and the risk of breast cancer. *Epidemiology*. 2007;18(2):266-269. doi:10.1097/01.ede.0000253934.58618.98 | shorting the data of incidence risk estimates of breast and gynecological cancer by antidepressant use |
| 8 | Busby J, Mills K, Zhang SD, Liberante FG, Cardwell CR. Selective serotonin reuptake inhibitor use and breast cancer survival: a population-based cohort study. *Breast Cancer Res*. | shorting the data of incidence risk estimates of breast and gynecological cancer by |

| | | |
|---|---|---|
| | 2018;20(1):4. Published 2018 Jan 19. doi:10.1186/s13058-017-0928-0 | antidepressant use |
| 9 | Ballou Y, Rivas A, Belmont A, et al. 5-HT serotonin receptors modulate mitogenic signaling and impact tumor cell viability. *Mol Clin Oncol*. 2018;9(3):243-254. doi:10.3892/mco.2018.1681 | shorting the data of incidence risk estimates of breast and gynecological cancer by antidepressant use |
| 10 | Azoulay L, Yin H, Renoux C, Suissa S. The use of atypical antipsychotics and the risk of breast cancer. *Breast Cancer Res Treat*. 2011;129(2):541-548. doi:10.1007/s10549-011-1506-2 | shorting the data of incidence risk estimates of breast and gynecological cancer by antidepressant use |
| 11 | Anthony HM, Kenny TE, MacKinnon AU. Drugs in the aetiology of cancer: a retrospective study. *Int J Epidemiol*. 1982;11(4):336-344. doi:10.1093/ije/11.4.336 | shorting the data of incidence risk estimates of breast and gynecological cancer by antidepressant use |
| 12 | Cohn E, Lurie I, Yang YX, et al. Posttraumatic Stress Disorder and Cancer Risk: A Nested Case-Control Study. *J Trauma Stress*. 2018;31(6):919-926. doi:10.1002/jts.22345 | shorting the data of incidence risk estimates of breast and gynecological cancer by antidepressant use |
| 13 | Friedman GD, Udaltsova N, Chan J, Quesenberry CP Jr, Habel LA. Screening pharmaceuticals for possible carcinogenic effects: initial positive results for drugs not previously screened. *Cancer Causes Control*. 2009;20(10):1821-1835. doi:10.1007/s10552-009-9375-2 | shorting the data of incidence risk estimates of breast and gynecological cancer by antidepressant use |
| 14 | Stebbing J, Powles T, Mandalia S, Nelson M, Gazzard B, Bower M. Use of antidepressants and risk of cancer in individuals infected with HIV. *J Clin Oncol*. 2008;26(14):2305-2310. doi:10.1200/JCO.2007.15.9681 | 1.1.1 shorting the data of incidence risk estimates of breast and gynecological cancer by antidepressant use for general population |
| 15 | Sun LM, Lin MC, Liang JA, et al. Does use of tetracyclic antidepressant-mirtazapine reduce cancer risk in depression patients?. *Pharmacoepidemiol Drug Saf*. 2013;22(12):1292-1297. doi:10.1002/pds.3523 | shorting the data of incidence risk estimates of breast and gynecological cancer by antidepressant use |
| 16 | Sun LM, Lin MC, Liang JA, et al. Does use of tetracyclic antidepressant-mirtazapine reduce cancer risk in depression patients?. *Pharmacoepidemiol Drug Saf*. 2013;22(12):1292- | shorting the data of incidence risk estimates of breast and gynecological cancer by |

| | | |
|---|---|---|
| | 1297. doi:10.1002/pds.3523 | antidepressant use |
| 17 | Huang T, Poole EM, Okereke OI, et al. Depression and risk of epithelial ovarian cancer: Results from two large prospective cohort studies. *Gynecol Oncol*. 2015;139(3):481-486. doi:10.1016/j.ygyno.2015.10.004 | shorting the data of incidence risk estimates of breast and gynecological cancer by antidepressant use |
| 18 | Lacey JV Jr, Sherman ME, Hartge P, Schatzkin A, Schairer C. Medication use and risk of ovarian carcinoma: a prospective study. *Int J Cancer*. 2004;108(2):281-286. doi:10.1002/ijc.11538 | shorting the data of incidence risk estimates of breast and gynecological cancer by antidepressant use |
| 19 | Ashbury JE, Lévesque LE, Beck PA, Aronson KJ. A population-based case-control study of Selective Serotonin Reuptake Inhibitors (SSRIs) and breast cancer: the impact of duration of use, cumulative dose and latency. *BMC Med*. 2010;8:90. Published 2010 Dec 22. doi:10.1186/1741-7015-8-90 | duplicate study data |

**Supplementary Figure 1. Dose-response for cumulative defined daily dose of antidepressant use and incidence risk of endometrial, corpus uteri and cervical cancer.**

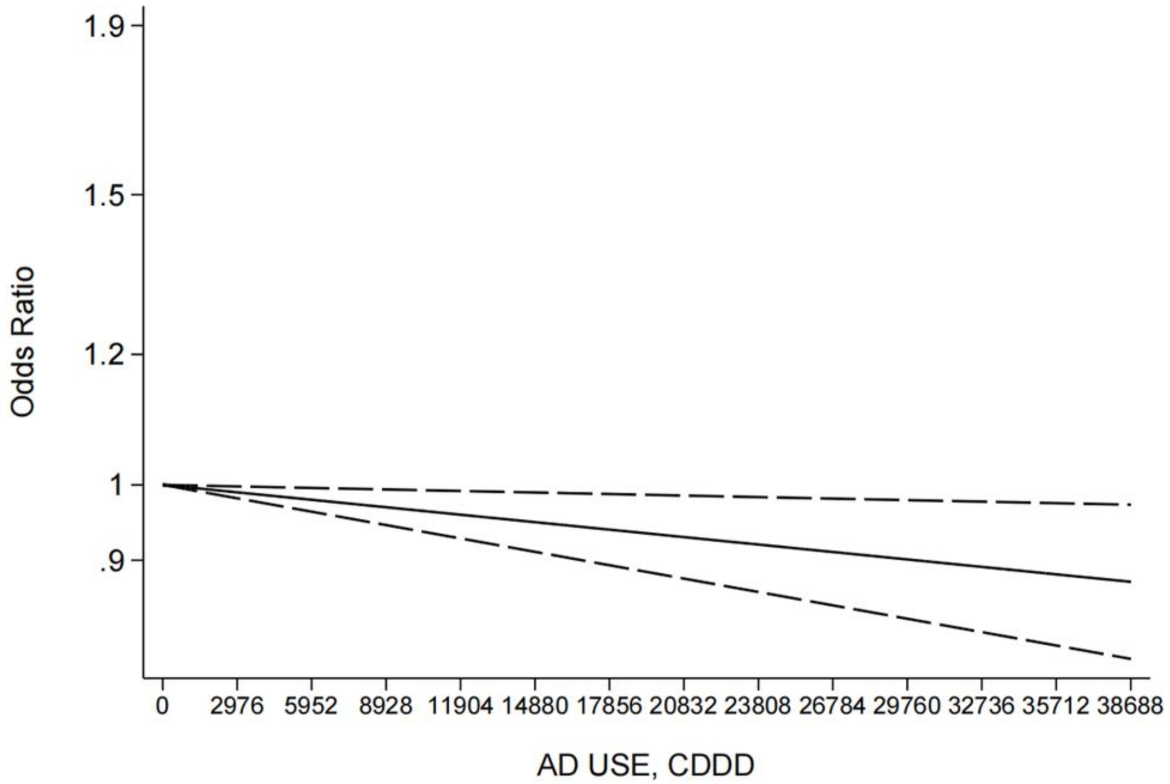


Abbreviations: AD: antidepressant; CDDD: cumulative defined daily dose.

The black solid line and the black long dashed line represent the estimated odds ratios (ORs) with corresponding 95% confidence intervals (CIs) for the non-linearity or the linearity.

**Supplementary Figure 2. Leave-one-out sensitivity analyses for the risk of breast and gynecological cancer**

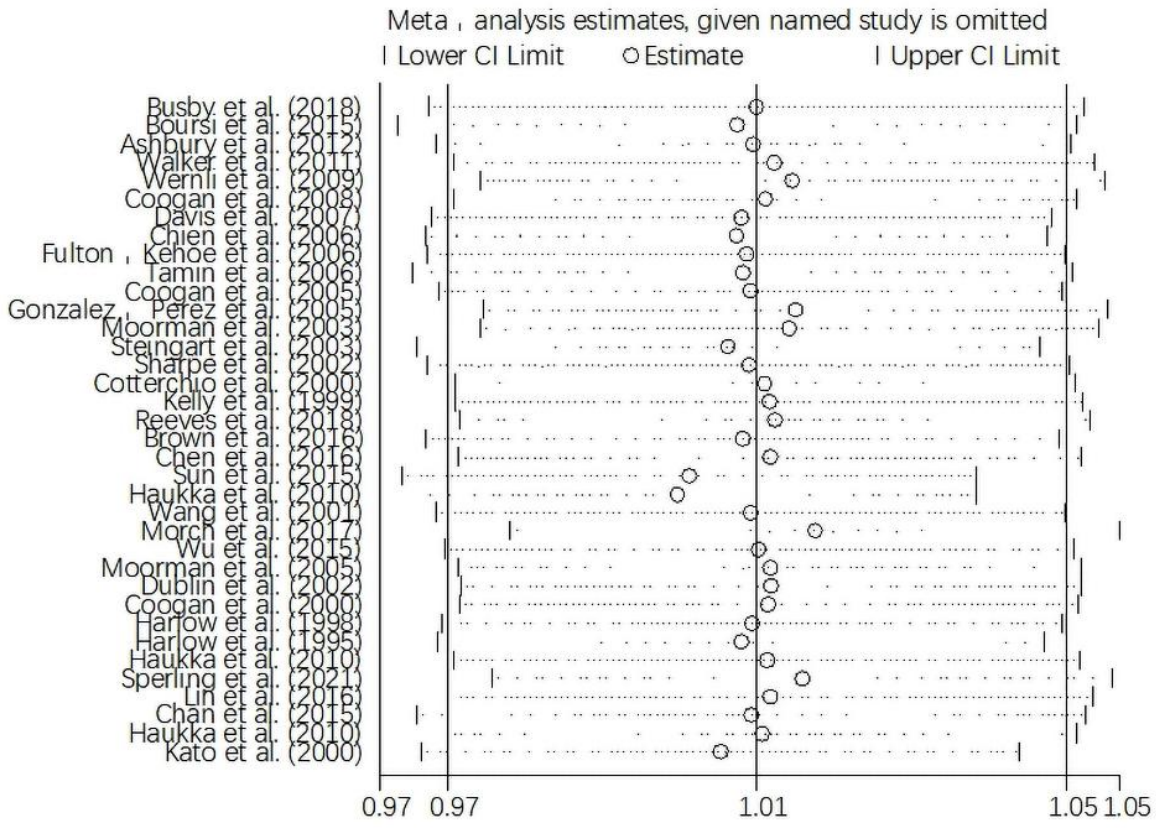

Supplement: Supplementary file 1 [file DataSheet_1.pdf]
